# Rational synthesis of benzimidazole[3]arenes by Cu^II^-catalyzed post-macrocyclization transformation†
†Dedicated to the late Mr Akihiro Mizoguchi for his contribution to the development of this study.
‡
‡Electronic supplementary information (ESI) available: Experimental details and characterization data. CCDC 1585798–1585799 and 1585801. For ESI and crystallographic data in CIF or other electronic format see DOI: 10.1039/c8sc03086c


**DOI:** 10.1039/c8sc03086c

**Published:** 2018-09-05

**Authors:** Shohei Tashiro, Tsutomu Umeki, Ryou Kubota, Mitsuhiko Shionoya

**Affiliations:** a Department of Chemistry , Graduate School of Science , The University of Tokyo , 7-3-1 Hongo , Bunkyo-ku , Tokyo 113-0033 , Japan . Email: shionoya@chem.s.u-tokyo.ac.jp

## Abstract

A new series of calix[*n*]arene analogues, benzimidazole[3]arenes, was rationally synthesized by Cu^II^-catalyzed post-macrocyclization transformation of a tris(*o*-phenylenediamine) macrocycle, and fully characterized by NMR, MS, and single-crystal X-ray diffraction (XRD) analyses. The resulting *syn*- and *anti*-benzimidazole[3]arenes have a bowl-shaped and a warped structure, respectively, in their crystalline states, and both display a dynamic inversion behavior in solution. This modification resulted in strong fluorescence due to the generated benzimidazole moieties. The mechanistic study of the post-macrocyclization transformation demonstrated that the formation of both benzimidazole[3]arenes was catalyzed, *via* triimine intermediates, by Cu^II^ ions in air through oxidation and cyclization of the tris(*o*-phenylenediamine) macrocycle.

## Abstract 2

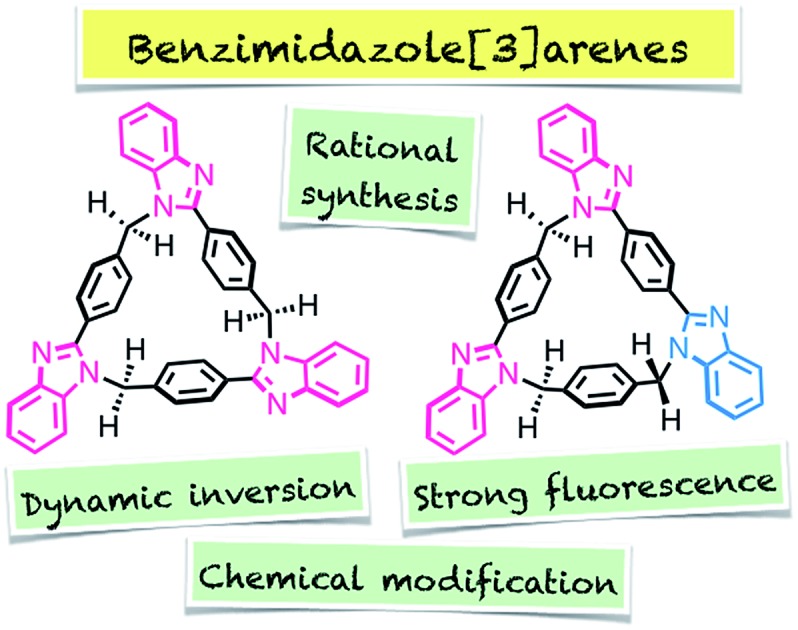
A new series of calix[*n*]arene analogues, benzimidazole[3]arenes, was rationally synthesized by Cu^II^-catalyzed post-macrocyclization transformation of a tris(*o*-phenylenediamine) macrocycle, and fully characterized by NMR, MS, and single-crystal X-ray diffraction (XRD) analyses.

## Introduction

Since the discovery of calix[4]arene and its analogues such as cyclotriveratrylene, pyrogallol[*n*]arenes, and resorcin[*n*]arenes,^1^ macrocyclic compounds with several aromatic rings circularly arranged through methylene linkers have attracted much attention in both fundamental and applied chemistry. Significant efforts have been devoted to the synthesis of their new series including pillar[*n*]arenes,2*a* pillar[*n*]quinones,2*b* asar[*n*]arenes,2*c* biphen[*n*]arenes,2*d* oxatub[*n*]anrenes,2*e* and others.2f–i These macrocycles have a well-defined hydrophobic cavity enclosed by a relatively flexible ring framework due to the presence of methylene linkers. It is also worth noting that some macrocycles are chiral when they have a circumferentially and axially anisotropic three-dimensional structure.^3^ Their structure and function depend heavily on the type and sequence feature of the building unit of the macrocycles.

N-Heterocycles are typical building blocks of macrocycles, as N-heterocycle-based calix[*n*]arene analogues such as calix[*n*]pyrroles,4*a* calix[*n*]imidazolium,4*b* calix[*n*]pyridines or pyridine[*n*]arenes,4c–e ExBox,4*f* Texas-sized box,4*g* and others4h–j exhibit strong fluorescence and ion recognition ability through non-covalent interactions. However, as far as we know, benzimidazole-based calix[*n*]arenes^5^,^6^ have not been reported so far due to the lack of rational synthetic strategies to circularly arrange benzimidazole moieties in macrocyclic skeletons, despite the fact that benzimidazole is known to show strong fluorescence, bioactivity, metal binding and ion recognition abilities.^7^ Here we report the Cu^II^-catalyzed facile synthesis, structures, and properties of two isomeric *syn*- and *anti*-benzimidazole[3]arenes (*syn*-**2** and *anti*-**3**) with *C*_3_- and *C*_1_-symmetry, respectively, based on the different permutations of three benzimidazole units. The molecular structures and their dynamic inversion behaviors were evaluated by single-crystal X-ray diffraction (XRD) and variable-temperature (VT) NMR analyses. We also found that three benzimidazole moieties in the macrocycle led to strong fluorescence.

**Fig. 1:**
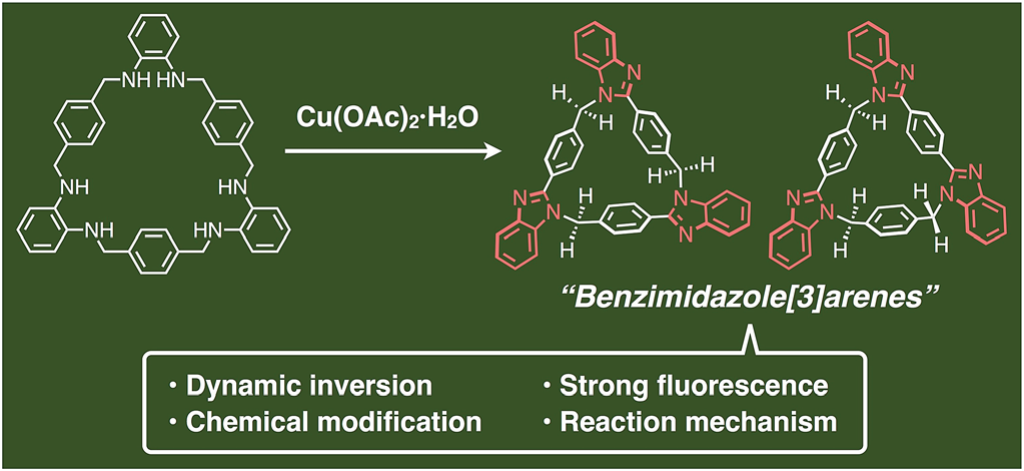
Schematic representation of the facile synthesis of benzimidazole[3]arenes through post-macrocyclization transformation.

A key step in the synthesis of *syn*-**2** and *anti*-**3** is post-macrocyclization transformation of macrocyclic tris(*o*-phenylenediamine) (**1**), which can be easily prepared from *o*-phenylenediamine and terephthalaldehyde in two steps of macrocyclization and hydrogenation.8*a* As in our previous report,^8^ macrocycle **1** reacted with Pd^II^ ions to form a trinuclear Pd^II^-macrocycle. However, we found that a reaction with Cu^II^ ions produced not trinuclear Cu^II^-macrocycles of **1**, but two isomeric *syn*- and *anti*-benzimidazole[3]arenes (*syn*-**2** and *anti*-**3**) through a Cu^II^-catalyzed transformation reaction (Fig. 1). In general, calix[*n*]arene and its analogues can be synthesized by simple macrocyclization of each building block.^1^,^2^ However, it is not always suitable to apply this direct macrocyclization to an unsymmetrical arrangement of building blocks for lower-symmetry macrocycles. In contrast, post-macrocyclization transformation^9^ is a powerful tool to prepare lower-symmetry macrocycles as proven by the intensive synthetic studies of diverse porphyrinoids, because a macrocyclic skeleton is asymmetrically folded by the macrocyclic transformation reaction.^10^

## Results and discussion

**Fig. 2:**
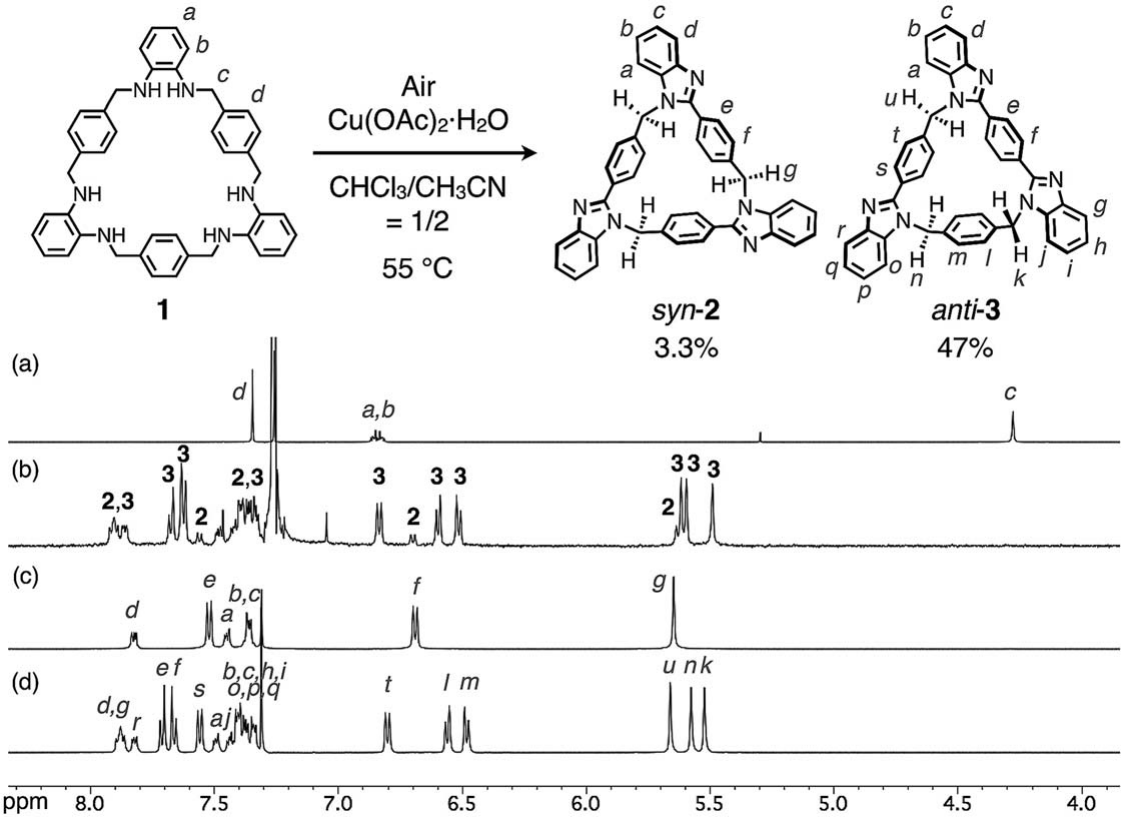
Reaction conditions of the post-macrocyclization transformation and ^1^H NMR spectra (500 MHz, 300 K, CDCl_3_ for (a) and (b) or CDCl_3_/CD_3_OD = 20/1 for (c) and (d)) of (a) **1**, (b) crude product, (c) *syn*-**2** and (d) *anti*-**3**.

Macrocycle **1** was reacted with an equimolar amount of Cu(OAc)_2_·H_2_O in a mixed solvent of CHCl_3_/CH_3_CN = 1/2 (v/v) at 55 °C in air ([**1**] = 5.0 mM). The reaction mixture became brown after 15 min, and then turned green after one day. The ^1^H NMR spectrum of the greenish reaction mixture showed broadened proton signals, indicating that Cu^II^ ions interacted with products. The ^1^H NMR analysis of the crude product after removing Cu^II^ ions by a liquid separating operation indicated that **1** was completely consumed to form the target compounds, *syn*- and *anti*-benzimidazole[3]arenes (*syn*-**2** and *anti*-**3**), in the molar ratio of *ca.* 1 : 10 (Fig. 2a and b). The mixture was separated by silica gel chromatography and then purified by recrystallization to afford *syn*-**2** and *anti*-**3** in 3.3% and 47% isolated yields, respectively (Fig. 2c and d). This selectivity is discussed later. The products were fully characterized by high-resolution electrospray ionization time-of-flight mass spectrometry (ESI-TOF MS) (*m*/*z* = 619.2593 and 619.2593 for [*syn*-**2** + H]^+^ and [*anti*-**3** + H]^+^, respectively), single-crystal X-ray diffraction (XRD), and NMR analyses as shown below.

**Fig. 3:**
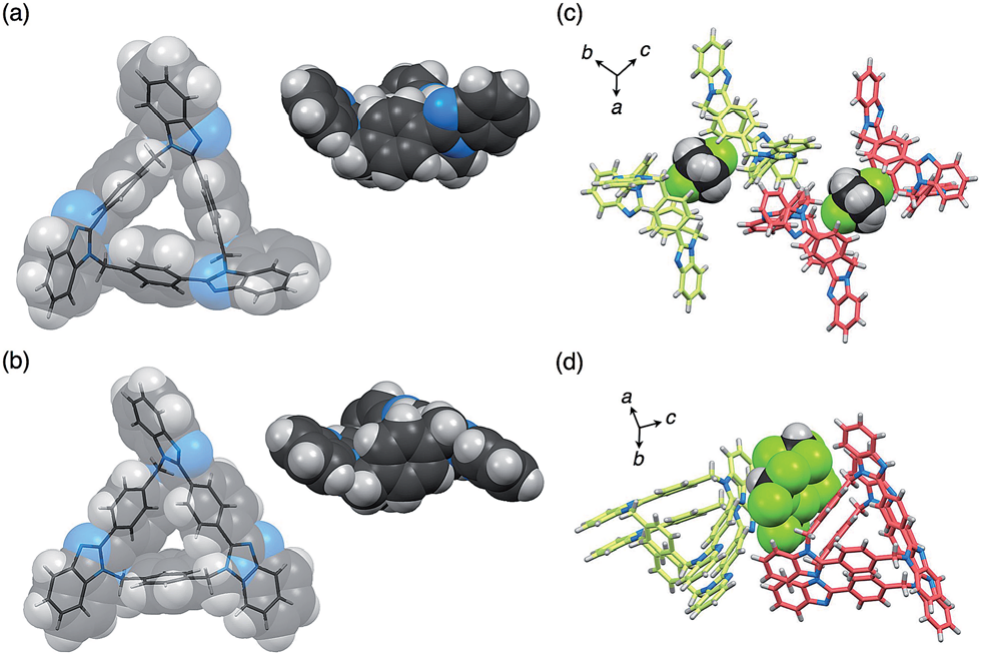
Molecular structures of (a) *syn*-**2** and (b) *anti*-**3** (top and side views), and crystal packing structures of (c) *syn*-**2** and (d) *anti*-**3**. In (c) and (d), macrocycles and solvents are represented by stick and space-filling models, respectively, and colors for the carbon atoms of (*P*)- and (*M*)-stereoisomers are yellow-green and pale-red, respectively.

The molecular structures of both compounds *syn*-**2** and *anti*-**3** have been determined by single-crystal XRD analyses. It was confirmed that both compounds are 21-membered macrocycles composed of three benzimidazole units. In the molecular structure of *syn*-**2**, three 2-phenylbenzimidazole moieties are symmetrically arranged in the same direction to form a *C*_3_-symmetry structure in which all three methylene linkers are oriented toward the convex face of a folded bowl-shaped conformation (Fig. 3a). On the other hand, two benzimidazole moieties of *anti*-**3** are directly connected to a *p*-phenylene moiety to form a 1,4-bis(benzimidazol-2-yl)benzene moiety with an extended conjugation system, which gives a warped *C*_1_-symmetry structure (Fig. 3b) in combination with the rest of the structure containing a 2-phenylbenzimidazole and a benzene moiety. It is worth noting here that both structures are chiral, as evident from the fact that both (*P*)- and (*M*)-stereoisomers coexist in the ratio of 1 to 1 in each crystal of *syn*-**2** and *anti*-**3**.^11^ For instance, two molecules of *syn*-**2** formed a homochiral dimer sandwiching one CH_2_Cl_2_ molecule between two small cavities of *syn*-**2**, and the other enantiomeric dimers were lined up in parallel (Fig. 3c). For *anti*-**3**, (*P*)- or (*M*)-stereoisomers of *anti*-**3** were stacked to form homochiral columns. The interstitial spaces formed between enantiomerically paired columns were occupied by CHCl_3_ molecules (Fig. 3d).

We next evaluated the solution-state structures of *syn*-**2** and *anti*-**3** in CDCl_3_ based on ^1^H NMR spectroscopy (Fig. 2c and d). First, the signals of some *p*-phenylene protons next to methylene groups were observed in the range from 6.5 to 6.8 ppm, significantly upfield shifted from typical *p*-phenylene signals. These characteristic *p*-phenylene signals suggest that the folded conformations of *syn*-**2** and *anti*-**3** observed in the crystal state, in which the *p*-phenylene protons face benzimidazole moieties, are maintained also in CDCl_3_. On the other hand, the ^1^H NMR spectra of *syn*-**2** and *anti*-**3** in CDCl_3_ indicate the formation of *C*_3h_- and *C*_s_-symmetry structures at 300 K, respectively. This higher symmetry in solution suggests that the rate of racemization of *syn*-**2** and *anti*-**3** between (*P*)- and (*M*)-isomers is significantly faster than the NMR timescale in CDCl_3_.

**Fig. 4:**
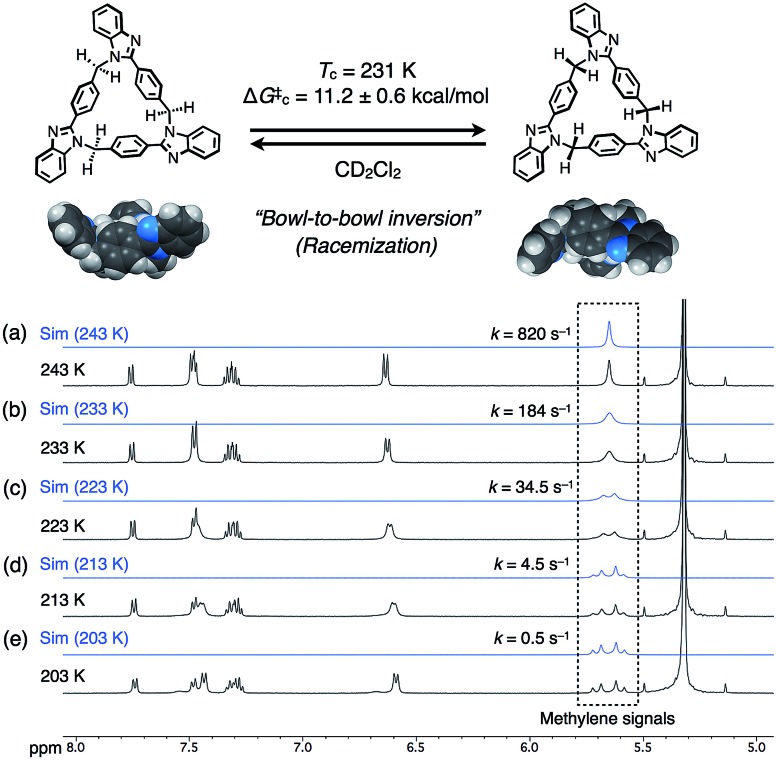
Schematic representation of the bowl-to-bowl inversion of *syn*-**2**, and simulated (blue lines, 5.5–5.8 ppm) and obtained (black lines, 5.0–8.0 ppm) ^1^H NMR spectra (500 MHz, CD_2_Cl_2_) of *syn*-**2** at (a) 243, (b) 233, (c) 223, (d) 213, and (e) 203 K.

The dynamic behaviors of *syn*-**2** and *anti*-**3** in solution were then examined by VT ^1^H NMR measurements in CD_2_Cl_2_. When the temperature of *syn*-**2** in CD_2_Cl_2_ was lowered to 203 K, the methylene signals around 5.65 ppm split into geminally coupled two doublet signals. In contrast, the other aromatic signals did not split except for the appearance of small broad signals probably due to the presence of a minor conformational isomer at 203 K (Fig. 4). The desymmetrization of only the methylene protons indicates that the temperature-dependent dynamic behavior is mainly derived from the racemization of (*P*)- and (*M*)-stereoisomers by the bowl-to-bowl inversion that is slower than the NMR timescale at 203 K. To estimate the coalescence temperature (*T*_c_) and inversion barrier Δ*G*‡c of *syn*-**2** at *T*_c_, the Eyring plot was drawn based on the dynamic ^1^H NMR line-shape simulation (Fig. S24‡). As a result, the *T*_c_ and Δ*G*‡c values were estimated to be 231 K and 11.2 ± 0.6 kcal mol^–1^, respectively, and Δ*H*^‡^ and Δ*S*^‡^ were also calculated to be 17.8 ± 0.3 kcal mol^–1^ and 28.5 ± 1.4 cal mol^–1^ K^–1^, respectively. Macrocycle *anti*-**3** also exhibited similar behaviors, and VT ^1^H NMR spectra were similarly analyzed to estimate *T*_c_, Δ*G*‡c, Δ*H*^‡^, and Δ*S*^‡^, which were found to be 230 K, 11.0 ± 0.4 kcal mol^–1^, 13.8 ± 0.2 kcal mol^–1^, and 12.3 ± 0.9 cal mol^–1^ K^–1^, respectively (Fig. S25 and S26‡). The resultant inversion barriers of *syn*-**2** (11.2 kcal mol^–1^) and *anti*-**3** (11.0 kcal mol^–1^) were found to be almost the same, and it should be noted that the inversion barriers are comparable with that of corannulene (11.5 kcal mol^–1^),^12^ which is a representative bowl-shaped hydrocarbon.

**Fig. 5:**
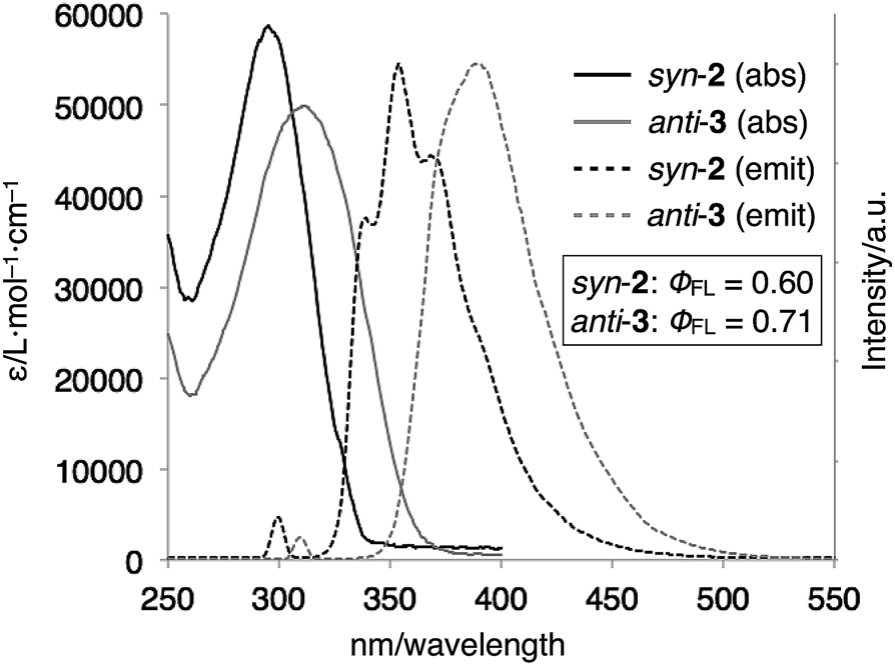
UV-vis absorption and normalized fluorescence spectra (CHCl_3_, 298 K) of *syn*-**2** (1.5 μM, *λ*_ex_ = 300 nm) and *anti*-**3** (3.0 μM, *λ*_ex_ = 310 nm).

The optical properties of *syn*-**2** and *anti*-**3** were examined by UV-vis absorption and fluorescence spectroscopies (Fig. 5). The UV-vis spectra of *syn*-**2** and *anti*-**3** in CDCl_3_ at 298 K showed absorption bands at *λ*_max_ = 296 nm and 312 nm, respectively. The red-shifted absorption of *anti*-**3** can be best explained by the presence of the 1,4-bis(benzimidazol-2-yl)benzene moiety with an extended conjugation system. This structural feature of *anti*-**3** also gave fluorescence at longer wavelength (*λ*_max_ = 390 nm) than that of *syn*-**2** (*λ*_max_ = 339, 354, 369 nm) in CHCl_3_ at 293 K. Quantum yields of *syn*-**2** and *anti*-**3** in degassed CHCl_3_ were determined to be 0.60 and 0.71, respectively, which are comparable to the quantum yield of 1-methyl-2-phenylbenzimidazole (*φ*_FL_ = 0.70), the structural unit of *syn*-**2** and *anti*-**3**.^13^

**Fig. 6:**
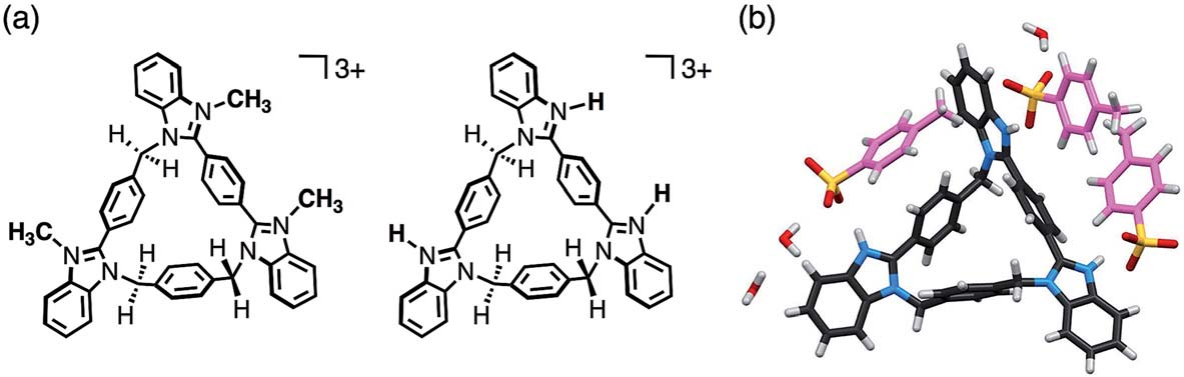
(a) Chemical structures of tri-methylated and tri-protonated *anti*-**3**. (b) Crystal structure of [H_3_(*anti*-**3**)(*p*-TsO)_3_]·(H_2_O)_3_. Disordered water molecules are omitted for clarity. The carbon atoms of *p*-TsO are shown in pink.

As benzimidazole[3]arenes possess three imidazole nitrogen atoms on the periphery of the macrocyclic skeletons, these nitrogen atoms were expected for further functionalization (Fig. 6a). For instance, *anti*-**3** underwent tri-methylation by reaction with CH_3_I, and the resulting tri-methylated product was found to be soluble in water, maintaining similar fluorescence properties (Fig. S27–S29‡). In addition, *anti*-**3** was fully protonated by mixing with *p*-toluenesulfonic acid monohydrate as confirmed by single-crystal XRD analysis. In the resultant crystal structure of [H_3_(*anti*-**3**)(*p*-TsO)_3_]·(H_2_O)_3_, all peripheral nitrogen atoms were protonated, interacting with *p*-TsO^–^ anions (Fig. 6b). The *p*-TsO^–^ anions further formed a hydrogen bonding network with water molecules along the *b*-axis. As a result, the crystal adopted a layer-by-layer structure composed of the macrocycle layers of *anti*-**3** and the hydrated layers of *p*-TsO^–^ anions (Fig. S32‡).

**Fig. 7:**
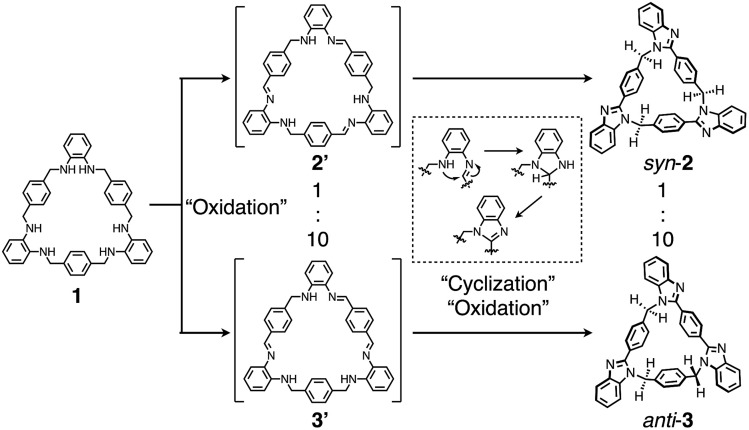
Plausible reaction mechanism of the formation of *syn*-**2** and *anti*-**3***via* intermediates **2′** and **3′**.

Finally, to discuss the reaction mechanism of this post-macrocyclization transformation, a diluted CD_3_CN solution of **1** (0.34 mM) and Cu(OAc)_2_·H_2_O (1.1 mM) was mixed at room temperature to monitor the reaction by ^1^H NMR measurements. We found that a yellow clear solution obtained after 20 min contained two different intermediates. Based on the chemical shifts, integral ratios, ^1^H–^1^H COSY, and NOESY correlations (Fig. S33–S35‡), the intermediates were most likely to be macrocyclic triimine compounds **2′** and **3′** (Fig. 7). For instance, singlet signals around 8.7–8.8 ppm and broad signals around 5.3–5.6 ppm can be assigned to imine and amine protons, respectively. The remaining methylene protons were also observed around 4.3–4.6 ppm. The signal patterns suggested that the main species was *C*_*s*_-symmetrical macrocyclic triimine **3′**, and the minor one appeared to be *C*_3*h*_-symmetrical triimine **2′**. The molar ratio of **2′** to **3′** was *ca.* 1 : 10, and further reaction of this solution for 8 days resulted in the formation of *syn*-**2** and *anti*-**3** in the molar ratio of *ca.* 1 : 10, which was almost the same as that under the synthetic conditions at 55 °C (Fig. 2b). This result indicated that the final ratio of *syn*-**2** to *anti*-**3** was determined at the first partial oxidation of **1** into triimine **2′** and **3′** (Fig. 7). The formation of the triimine intermediates was also confirmed by the ESI-TOF mass spectrometry measurement of a similarly prepared CH_3_CN reaction solution after 20 min (*m*/*z* = 647.26 for [**1**–6H + Na]^+^) (Fig. S36–S38‡).

The molar ratio of the formation of **2′** and **3′** (*ca.* 1 : 10) was more biased than the statistical ratio of 1 : 3. The more biased formation of **3′** is possibly attributed to the superiority in the kinetic and/or thermodynamic stability due to its extended conjugated structure. In the next reaction, the resultant imine carbons are subject to cyclization by the attack of the neighboring amino groups, followed by oxidative aromatization to afford benzimidazole moieties (Fig. 7). On the other hand, predominant formation of *syn*-**2** was not observed by changing the amount of Cu(OAc)_2_·H_2_O, reaction temperature, or solvent ratio as far as we examined.

In a plausible reaction mechanism shown in Fig. 7, Cu(OAc)_2_·H_2_O serves as an oxidant and a Lewis acid to facilitate several steps including oxidation to imine, cyclization, and oxidative aromatization. Because the conversion from **1** to *syn*-**2** or *anti*-**3** is a twelve-electron oxidation reaction, an equimolar amount of Cu(OAc)_2_·H_2_O should work as the catalyst in air. Moreover, we found that this reaction proceeded at higher temperature without Cu(OAc)_2_·H_2_O. When a solution of **1** in CD_3_CN was heated at 180 °C under microwave irradiation, *syn*-**2** and *anti*-**3** were formed after 105 min with some impurities (Fig. S39‡). Another oxidant, 2,3-dichloro-5,6-dicyano-*p*-benzoquinone (DDQ) (12 equiv.), also converted **1** into *syn*-**2** and *anti*-**3**, but a substantial amount of byproducts was also formed (Fig. S40‡). These results clearly indicate that O_2_ in air is the oxidant and that Cu(OAc)_2_·H_2_O catalyzes both the oxidation and cyclization steps to cleanly promote this reaction.

## Conclusions

In summary, two isomeric benzimidazole[3]arenes *syn*-**2** and *anti*-**3** have been rationally synthesized as a new series of calix[*n*]arene analogues by the Cu^II^-catalyzed post-macrocyclization transformation reaction of **1***via* two triimine intermediates **2′** and **3′**. The *C*_3_- and *C*_1_-symmetrical macrocyclic structures and their dynamic inversion behavior in solution were revealed by single-crystal XRD and VT NMR analyses, respectively. The three benzimidazole moieties also led to strong fluorescence and allowed chemical modification at the macrocyclic periphery. As another potential use of benzimidazole[3]arenes, study on metal complexation is in progress, and the resultant supramolecular structures will be reported elsewhere as separate studies. The present rational synthetic method and the potential applications are thus expected to make benzimidazole[3]arenes versatile as with calix[*n*]arenes and pillar[*n*]arenes that are being utilized around the world.

## Conflicts of interest

There are no conflicts to declare.

## Supplementary Material

Supplementary information

Crystal structure data
