# Supplementary material for: Rational synthesis of benzimidazole[3]arenes by CuII-catalyzed post-macrocyclization transformation
Source: Chem Sci. 2018 Sep 5;9(39):7614–9. doi: 10.1039/c8sc03086c (PMC6187690; doi:10.1039/c8sc03086c)
Supplement: Supplementary file 1 [file SC-009-C8SC03086C-s001.pdf]

Electronic Supplementary Material (ESI) for Chemical Science.
This journal is © The Royal Society of Chemistry 2018

Electronic Supplementary Information

Rational Synthesis of Benzimidazole[3]arenes by Cu^{II}-catalyzed Post-macrocyclization Transformation

Shohei Tashiro,* Tsutomu Umeki, Ryou Kubota, and Mitsuhiko Shionoya*

Department of Chemistry, Graduate School of Science, The University of Tokyo, 7-3-1 Hongo, Bunkyo-ku, Tokyo 113-0033, Japan

• **Materials and methods** page S2

• **Synthesis of *syn*-2 and *anti*-3** page S3

• **Crystal data of *syn*-2 and *anti*-3** page S10

• **Variable-temperature ^{1}H NMR of *syn*-2 and *anti*-3** page S13

• **Methylation of *anti*-3** page S14

• **Protonation of *anti*-3** page S16

• **Formation of triimine intermediates 2' and 3'** page S17

• **Reaction at higher temperature without $Cu(OAc)_2 \cdot H_2O$** page S22

• **Reaction with DDQ** page S23

• **References** page S23

Materials and methods

The synthesis of tris(*o*-phenylenediamine) macrocycle **1** was previously reported by us.[1] Other solvents, organic and inorganic reagents are commercially available, and were used without further purification. Column chromatography was performed using Merck Silica Gel 60 (230–400 mesh). Preparative TLC was conducted using Merck PLC Silica Gel 60 F_{254}.

NMR spectroscopic measurements were performed using a Bruker AVANCE 500 spectrometer. NMR spectra are calibrated as below; $CDCl_3$: $Si(CH_3)_4$ = 0 ppm for ^1H, $CDCl_3/CD_3OD$ = 20/1: $Si(CH_3)_4$ = 0 ppm for ^1H, $Si(CH_3)_4$ = 0 ppm for ^{13}C, CD_3CN: CHD_2CN = 1.94 ppm for ^1H, CD_2Cl_2: $CHDCl_2$ = 5.32 ppm for ^1H. The multiplicity of each signal for ^1H NMR was indicated by s (singlet), d (doublet), and m (multiplet). Measurements of the rate constants (k) for the inversion process of *syn*-**2** and *anti*-**3** were carried out by dynamic ^1H NMR line-shape simulation using a program iNMR (version 5.4.2). Eyring plots were generated using the rate constants (k) determined by dynamic ^1H NMR line-shape simulations. ESI-TOF mass spectra were recorded on a Waters LCT Premier XE spectrometer, in which HRMS data were collected using leucine enkephalin as an internal standard. UV-vis spectroscopy was performed using a HITACHI U-3500 spectrophotometer. The molar extinction coefficients of *syn*-**2** and *anti*-**3** are the averaged values of several measurements. Fluorescence spectra were measured with a HITACHI F-4500 fluorescence spectrophotometer. Photoluminescence (PL) quantum yields were determined with a Hamamatsu C9920-02G system for absolute PL quantum yield measurement equipped with an integrating sphere, and each $CHCl_3$ solution was degassed by Ar bubbling for 3 min in advance. The reproducibility of PL quantum yields was confirmed by multiple measurements. Melting points were measured using Yanaco MP-500D apparatus. Single-crystal XRD analyses were performed using a Rigaku RAXIS-RAPID imaging plate diffractometer with Mo$K\alpha$ radiation for *syn*-**2** and *anti*-**3** or a Rigaku XtaLAB P200 diffractometer with Cu$K\alpha$ radiation for protonated *anti*-**3**, and the obtained data were analyzed using a CrystalStructure crystallographic software package except for refinement, which was performed using SHELXL-2013 program suite.[2] X-ray structures were displayed using a Mercury program. Microwave irradiation experiments were performed using a Monowave 300 single-mode microwave reactor (Anton Paar GmbH). The reaction temperature was monitored with an external IR sensor and an internal fiber-optic temperature probe (ruby thermometer). Magnetron output power was controlled with the fiber-optic probe.

Synthesis of *syn*-2 and *anti*-3

Tris(*o*-phenylenediamine) macrocycle **1** (112 mg, 0.177 mmol, 1 equiv) was dissolved in a mixed solution of $CHCl_3$ (12 mL) and CH_3CN (16 mL), and the resultant yellow solution was then warmed to 55 °C with an oil bath. To the hot solution was added a blue solution of $Cu(OAc)_2 \cdot H_2O$ (35.3 mg, 0.177 mmol, 1 equiv) dissolved in CH_3CN (7 mL). The mixed solution immediately turned orange and was stirred at 55 °C for 24 h under air. During the reaction, the solution color turned from orange into brown, and eventually into green (Figure S1). After cooling the solution, it was evaporated at room temperature to give a yellowish green solid, which was then dissolved in $CHCl_3$ (30 mL). The green solution was washed with saturated aqueous NH_4Cl twice (20 mL × 2), and the aqueous layer was extracted with $CHCl_3$ twice (40, 20 mL). The combined organic layer was washed with water (50 mL), dried over anhydrous Na_2SO_4, and evaporated to afford a yellow solid (122 mg) as a crude product (^{1}H NMR: Figure S2). This material was purified by silica gel column chromatography ($CHCl_3/CH_3CN$ = 1/1 to $CHCl_3/CH_3OH$ = 20/1) and preparative thin-layer chromatography ($CHCl_3/CH_3CN$ = 1/1). Note that preparative TLC surpassed column chromatography in the separation of *syn*-**2** and *anti*-**3** as far as we examined. Roughly isolated products *syn*-**2** and *anti*-**3** were further purified by recrystallization. For instance, compound *syn*-**2** was recrystallized by vapor diffusion of water into a solution of *syn*-**2** (CH_2Cl_2/CH_3CN = ca. 3/1) to afford colorless blocks (3.6 mg, 5.8 μmol, 3.3%). On the other hand, compound *anti*-**3** was recrystallized by vapor diffusion of acetone into a $CHCl_3$ solution of *anti*-**3** to afford a colorless powder (52.2 mg, 84 μmol, 47%). The relatively low isolated yields of *syn*-**2** and *anti*-**3** are due to difficulty in the chromatographic separation of both isomers with similar polarity and the loss in the recrystallization processes.

syn-**2**; Mp: > 300 °C. ^{1}H NMR (500 MHz, $CDCl_3/CD_3OD$ = 20/1, 300 K): δ = 7.82 (m, 1H), 7.52 (d, J = 8.0 Hz, 2H), 7.45 (m, 1H), 7.36 (m, 2H), 6.69 (d, J = 8.0 Hz, 2H), 5.65 (s, 2H). ^{13}C NMR (126 MHz, $CDCl_3/CD_3OD$ = 20/1, 300 K): δ = 152.2, 142.2, 137.1, 136.4, 129.4, 128.8, 125.6, 123.7, 123.2, 119.8, 109.4, 47.1. UV-vis ($CHCl_3$, 298 K, [*syn*-**2**] = 1.5 μM): λ_{max} (nm) ($\varepsilon \times 10^{-4}$ (M^{-1} cm^{-1})) = 296 (6.0 ± 0.5). Fluorescence ($CHCl_3$, 298 K, λ_{ex} = 300 nm, [*syn*-**2**] = 1.5 μM): λ_{max} (nm) = 339, 354, 369. PL quantum yield ($CHCl_3$, rt, λ_{ex} = 300 nm, [*syn*-**2**] = 1 μM): ϕ = 0.60. HRMS (ESI-TOF): m/z = 619.2593 as $[C_{42}H_{30}N_6 + H]^+$ (calcd 619.2610).

anti-**3**; Mp: > 300 °C (when the solid was gradually heated). However, when the solid was quickly heated to *ca.* 270 °C, the solid melted. This may indicate that a phase transition occurred during slow heating. ^{1}H NMR (500 MHz, $CDCl_3/CD_3OD$ = 20/1, 300 K): δ = 7.88 (m, 2H), 7.82 (m, 1H), 7.71 (d, J = 8.0 Hz, 2H), 7.66 (d, J = 8.5 Hz, 2H), 7.56 (d, J = 8.0 Hz, 2H), 7.49 (m, 1H), 7.44 (m, 1H), 7.41 (m, 3H), 7.38 (m, 2H), 7.34 (m, 2H), 6.80 (d, J = 8.0 Hz, 2H), 6.56 (d, J = 8.0 Hz, 2H), 6.48 (d, J = 8.0 Hz, 2H), 5.66 (s, 2H), 5.58 (s, 2H), 5.52 (s, 2H). ^{13}C NMR (126 MHz, $CDCl_3/CD_3OD$ = 20/1, 300 K): δ = 152.3, 152.3, 152.2, 142.4, 142.4, 142.3, 137.9, 136.9, 136.8,

136.5, 135.6, 134.9, 131.1, 131.1, 129.7, 128.9, 128.7, 128.7, 126.0, 125.8, 125.7, 124.0, 123.8, 123.6, 123.4, 123.3, 123.2, 120.0, 120.0, 119.8, 109.6, 109.4, 109.4, 47.6, 47.5, 47.1. UV-vis ($CHCl_3$, 298 K, [*anti*-**3**] = 3 μM): λ_{max} (nm) ($\varepsilon \times 10^{-4}$ (M^{-1} cm^{-1})) = 312 (5.1 ± 0.1). Fluorescence ($CHCl_3$, 298 K, λ_{ex} = 310 nm, [**3**] = 3 μM): λ_{max} (nm) = 390. PL quantum yield ($CHCl_3$, rt, λ_{ex} = 310 nm, [*anti*-**3**] = 0.7 μM): ϕ = 0.71. HRMS (ESI-TOF): *m*/*z* = 619.2593 as $[C_{42}H_{30}N_6 + H]^+$ (calcd 619.2610).

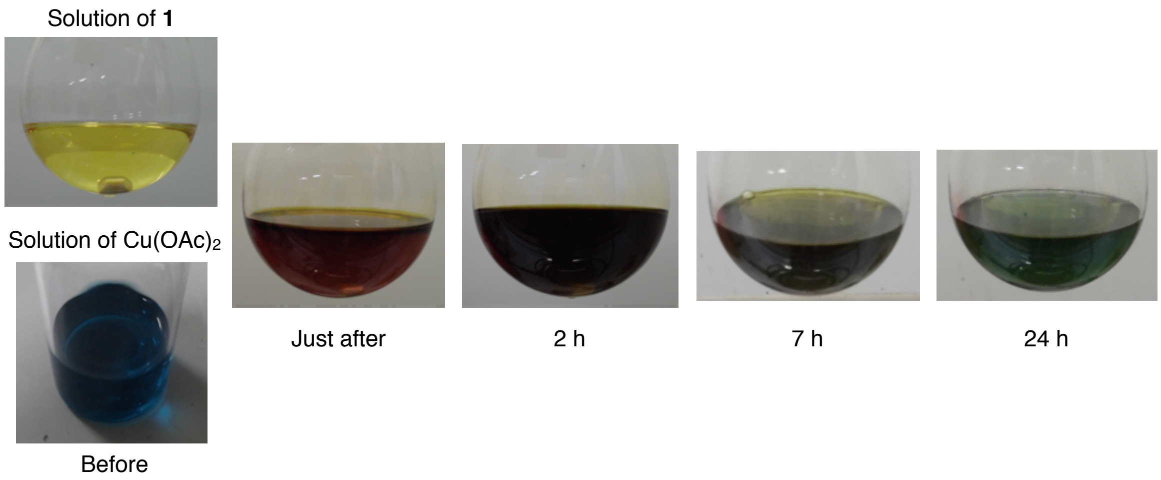

Figure S1. The color changes of the reaction solution.

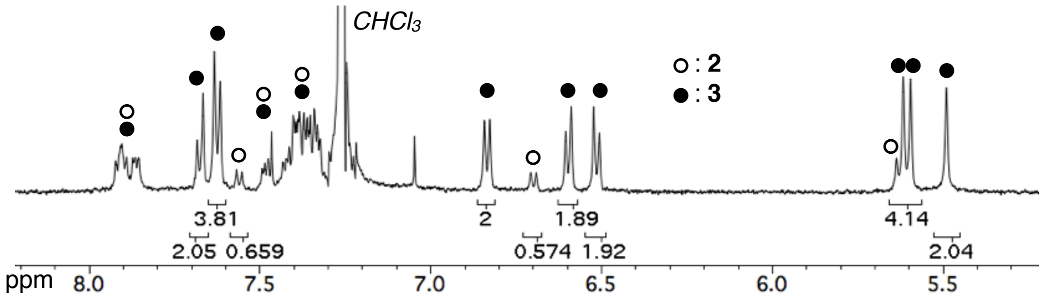

Figure S2. Partial ^{1}H NMR spectrum of the crude product (500 MHz, $CDCl_3$, 300 K).

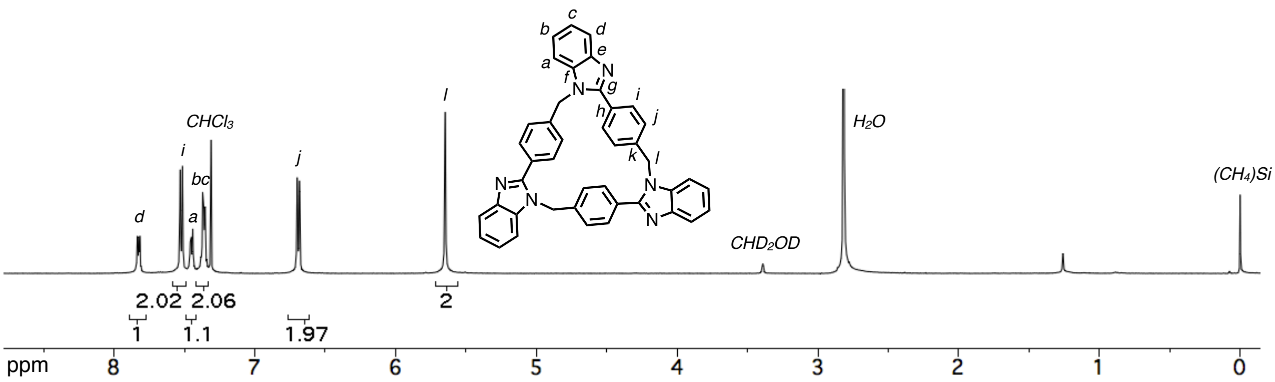

Figure S3. ^{1}H NMR spectrum of **2** (500 MHz, $CDCl_3/CD_3OD$ = 20/1, 300 K).

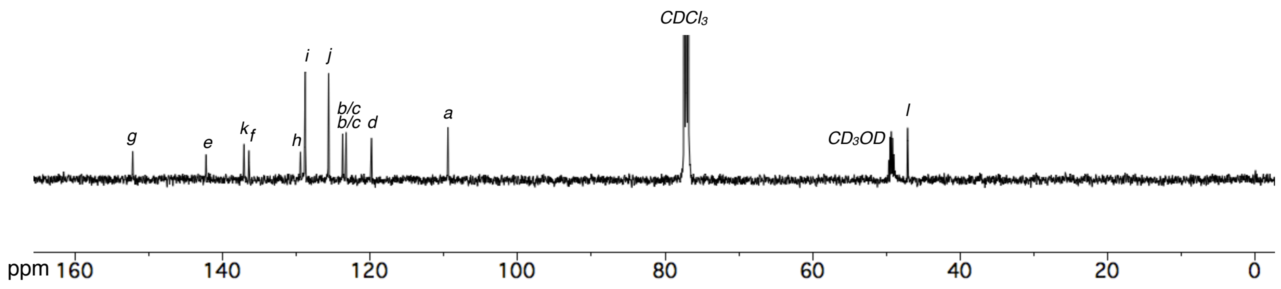

Figure S4. ^{13}C NMR spectrum of *syn*-**2** (126 MHz, $CDCl_3/CD_3OD$ = 20/1, 300 K).

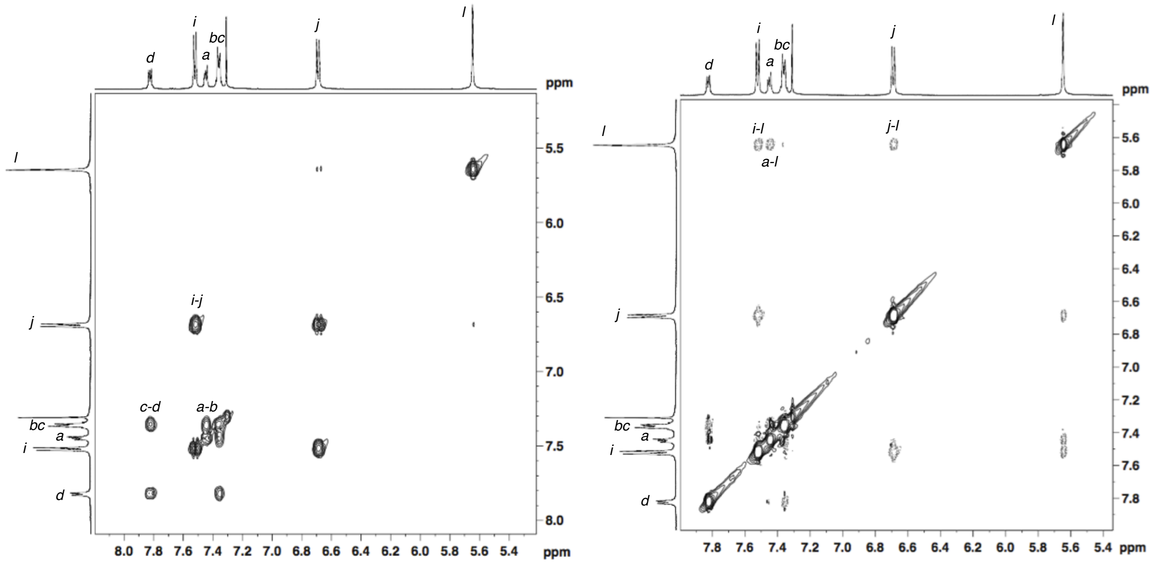

Figure S5. ^{1}H-^{1}H COSY (left) and ^{1}H-^{1}H NOESY (right, mixing time = 0.65 s) of *syn*-**2** (500 MHz, $CDCl_3/CD_3OD$ = 20/1, 300 K).

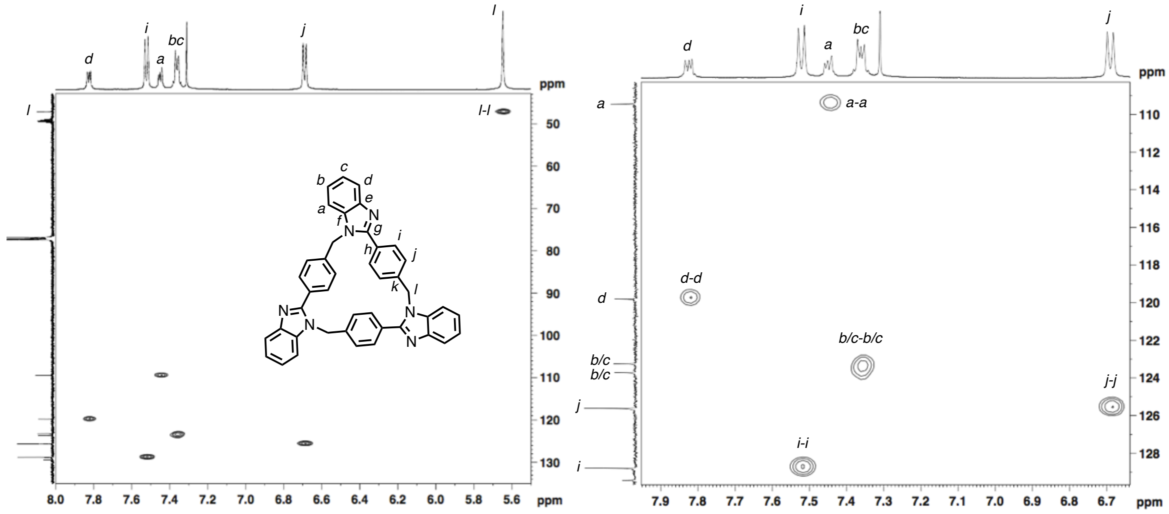

Figure S6. HSQC of *syn*-**2** (^{1}H: 500 MHz, ^{13}C: 126 MHz, $CDCl_3/CD_3OD$ = 20/1, 300 K).

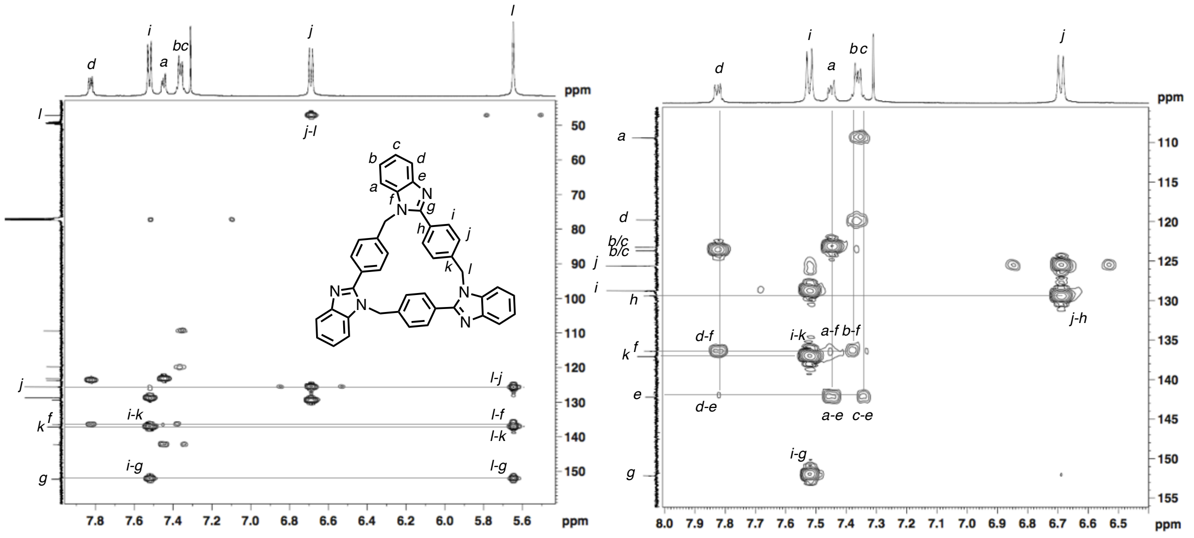

Figure S7. HMBC of *syn*-**2** (^{1}H: 500 MHz, ^{13}C: 126 MHz, $CDCl_3/CD_3OD$ = 20/1, 300 K).

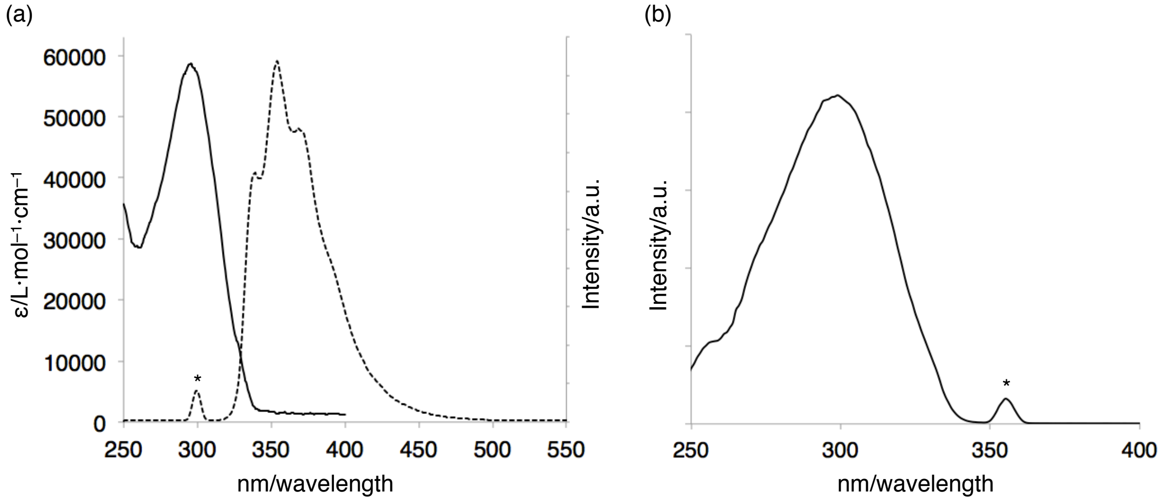

Figure S8. (a) UV-vis absorption (solid line) and luminescence (dash line) spectra of *syn*-**2** ($CHCl_3$, 298 K, [*syn*-**2**] = 1.5 μM, λ_{ex} = 300 nm) and (b) excitation spectrum of *syn*-**2** ($CHCl_3$, 298 K, [*syn*-**2**] = 1.5 μM, λ_{em} = 354 nm). The asterisk marks (*) indicate excitation bands.

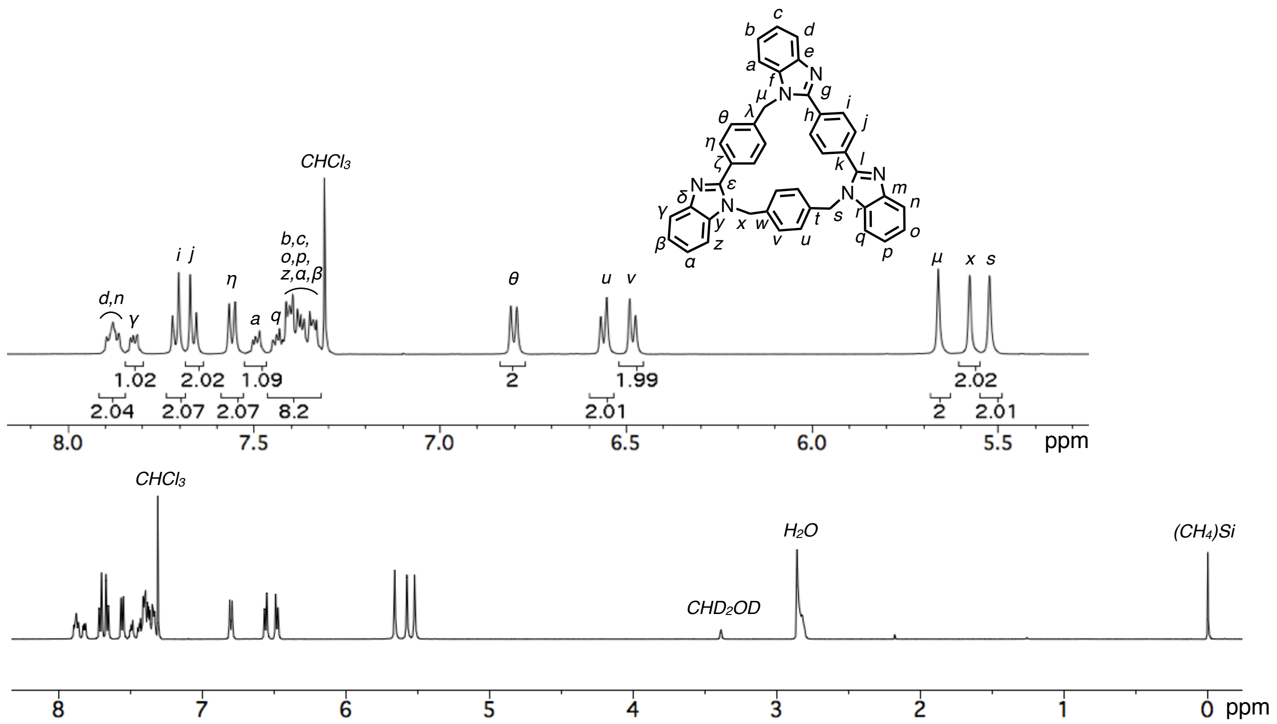

Figure S9. ^{1}H NMR spectra of *anti*-**3** (500 MHz, $CDCl_3/CD_3OD$ = 20/1, 300 K).

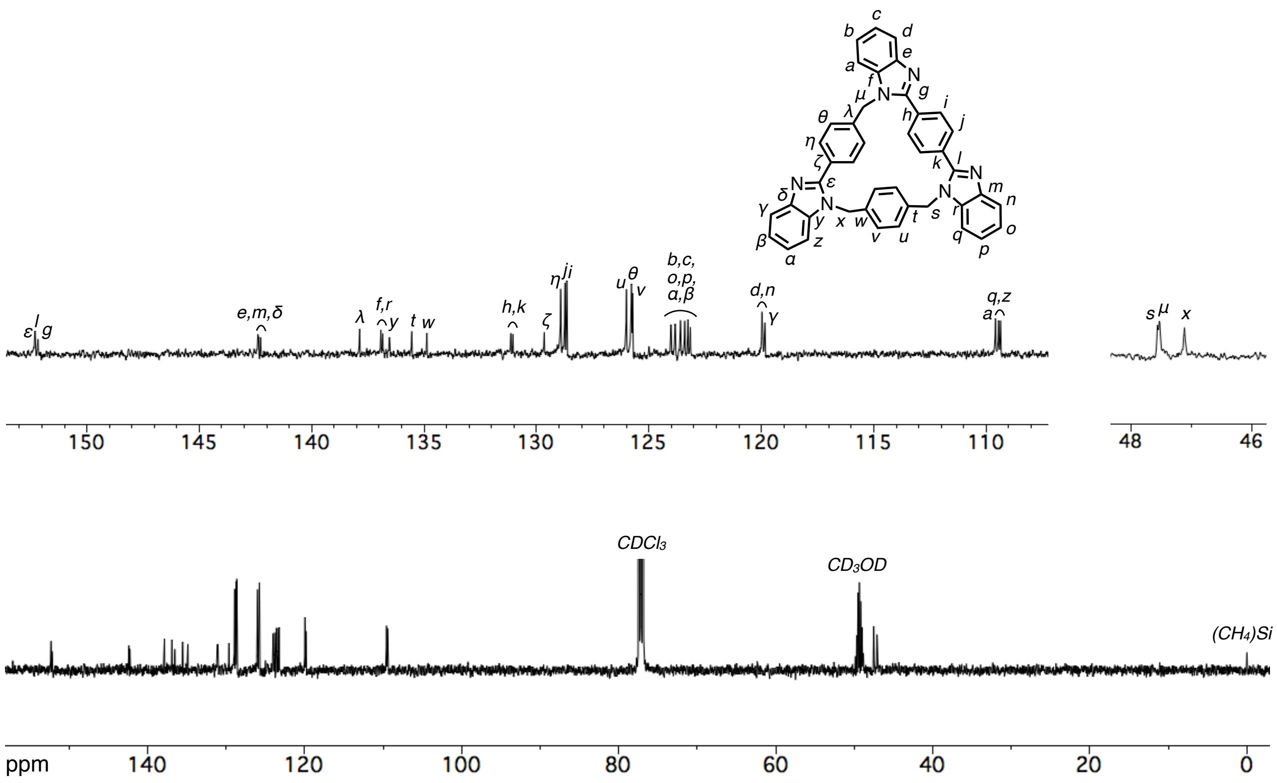

Figure S10. ^{13}C NMR spectra of *anti*-**3** (126 MHz, $CDCl_3/CD_3OD$ = 20/1, 300 K).

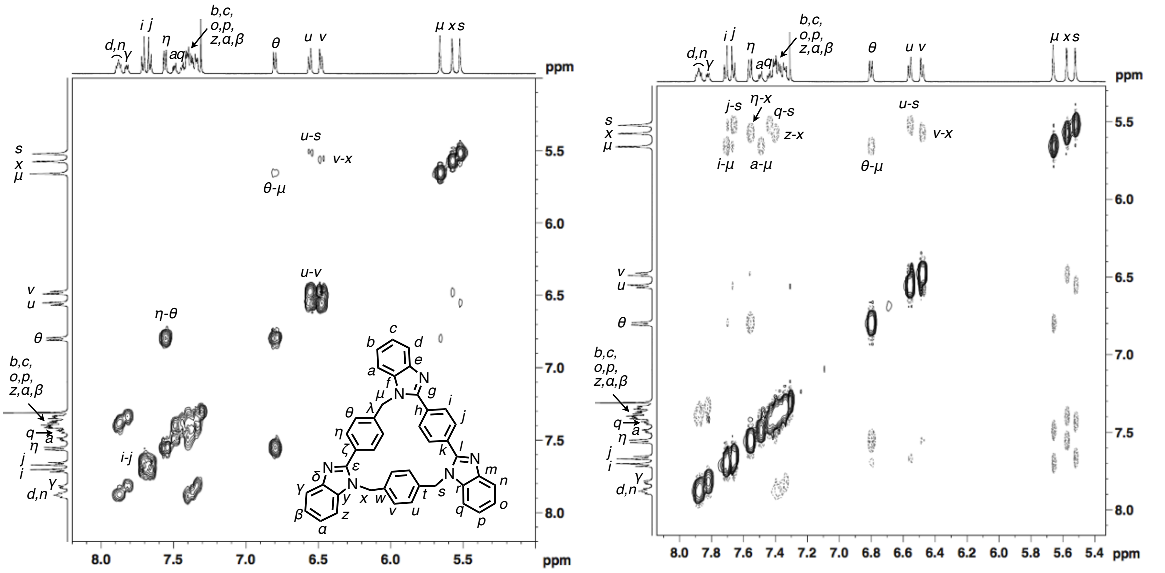

Figure S11. ^{1}H-^{1}H COSY (left) and ^{1}H-^{1}H NOESY (right, mixing time = 0.65 s) of *anti*-**3** (500 MHz, $CDCl_3/CD_3OD$ = 20/1, 300 K).

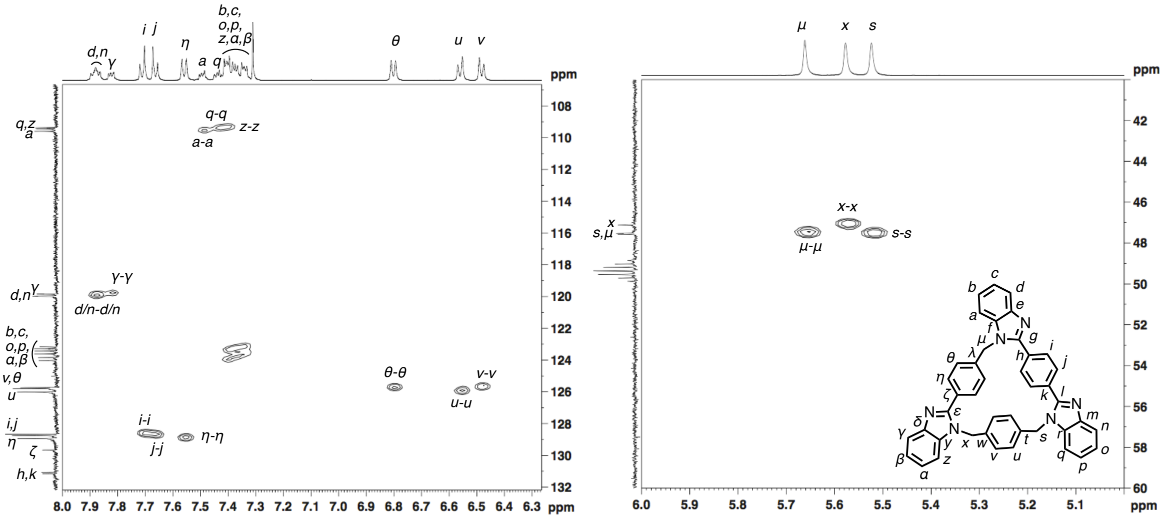

Figure S12. HSQC of *anti*-**3** (^{1}H: 500 MHz, ^{13}C: 126 MHz, $CDCl_3/CD_3OD$ = 20/1, 300 K).

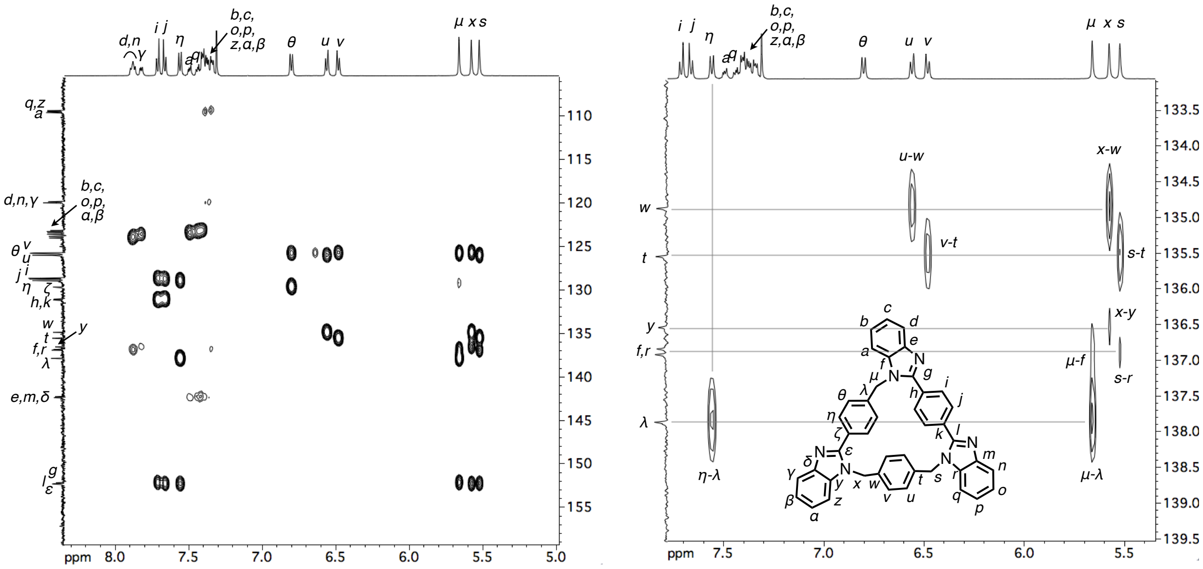

Figure S13. HMBC of *anti*-**3** (^{1}H: 500 MHz, ^{13}C: 126 MHz, $CDCl_3/CD_3OD$ = 20/1, 300 K).

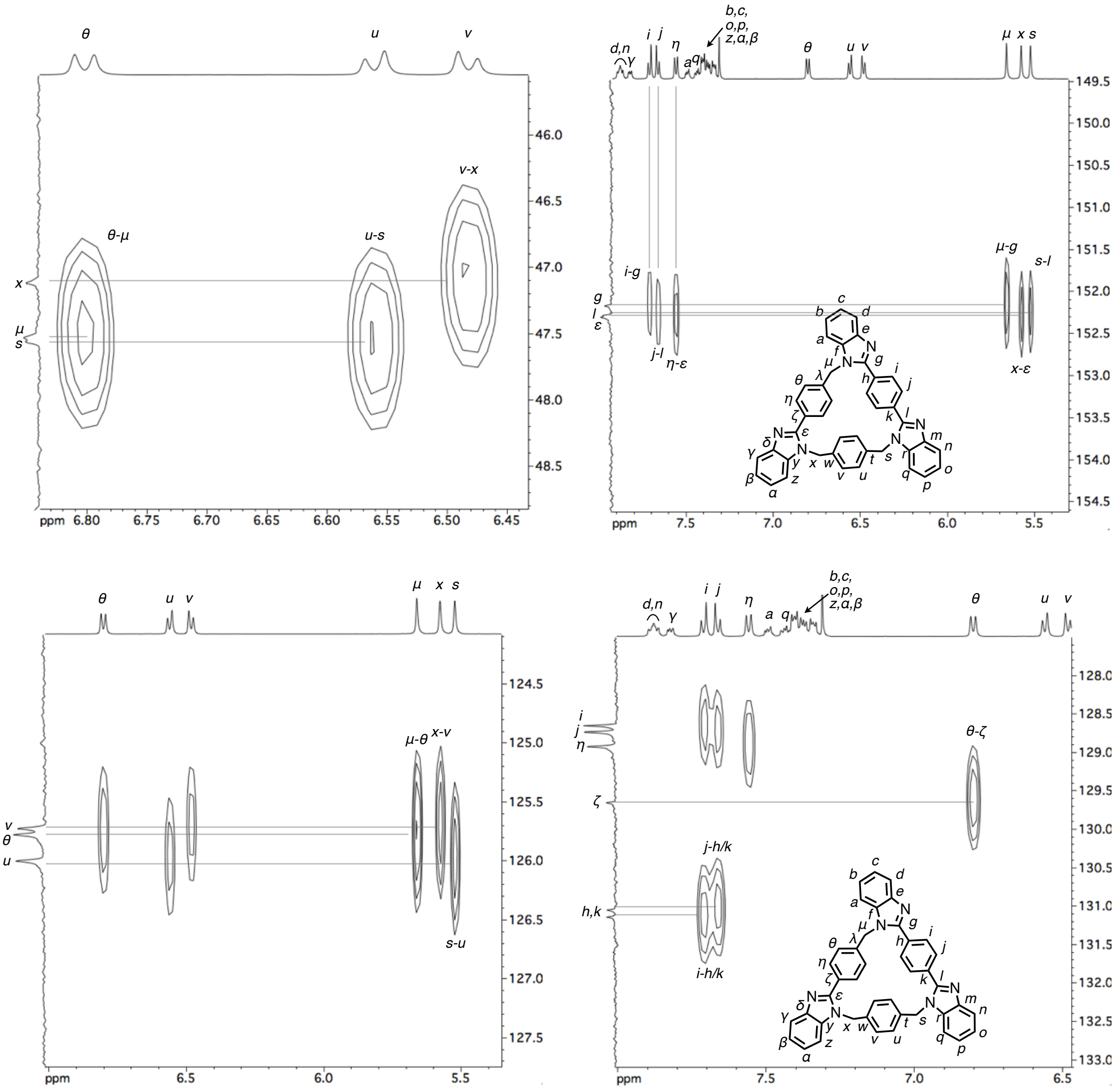

Figure S14. HMBC of *anti*-**3** (^{1}H: 500 MHz, ^{13}C: 126 MHz, $CDCl_3/CD_3OD$ = 20/1, 300 K).

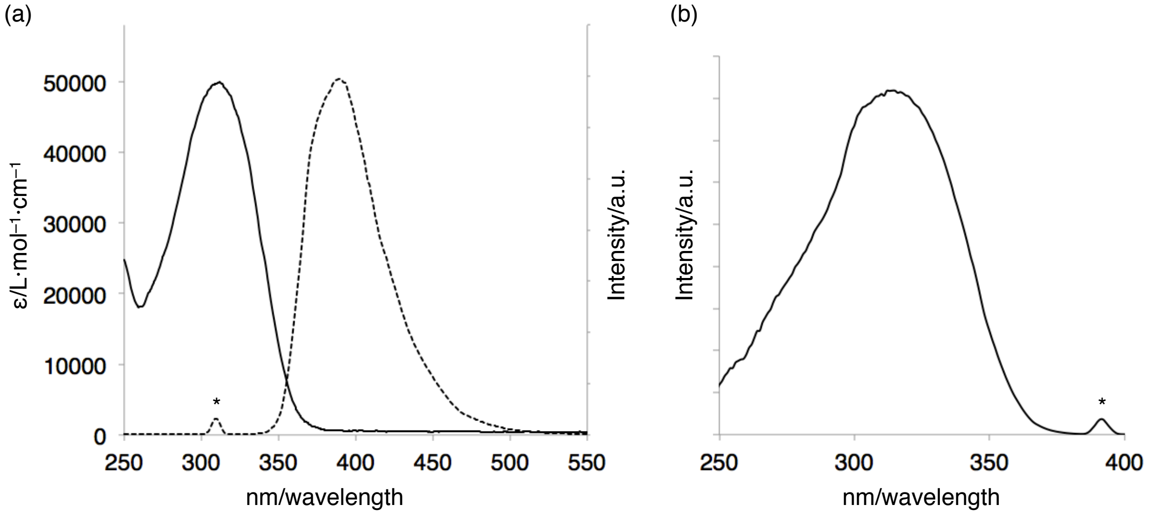

Figure S15. (a) UV-vis absorption (solid line) and luminescence (dash line) spectra of *anti*-**3** ($CHCl_3$, 298 K, [*anti*-**3**] = 3 μM, λ_{ex} = 310 nm) and (b) excitation spectrum of *anti*-**3** ($CHCl_3$, 298 K, [*anti*-**3**] = 3 μM, λ_{em} = 390 nm). The asterisk marks (*) indicate excitation bands.

Crystal data of *syn*-2 and *anti*-3

Pale yellow crystals of *syn*-**2** suitable for the single-crystal XRD analysis were obtained by slow evaporation of a CH_3CN/CH_2Cl_2 mixed solution of *syn*-**2**.

Crystal data for *syn*-**2**$\cdot(CH_2Cl_2)_2\cdot(H_2O)_{1.5}$: $C_{44}H_{37}Cl_4N_6O_{1.50}$, F_w = 815.59, crystal dimensions 0.50 × 0.30 × 0.25 mm, trigonal, space group R–3c, a = 16.5210(5), c = 49.1998(17) Å, V = 11629.6(8) Å^3, Z = 12, ρ_{calcd} = 1.397 g cm^{-3}, μ = 0.352 mm^{-1}, T = 93 K, λ(MoKα) = 0.71075 Å, $2\theta_{max}$ = 55.0°, 34693/2970 reflections collected/unique (R_{int} = 0.0311), R_1 = 0.0486 ($I > 2\sigma(I)$), wR_2 = 0.1371 (for all data), GOF = 1.057, largest diff. peak and hole 0.554/–1.304 eÅ^{-3}. CCDC deposit number 1585798.

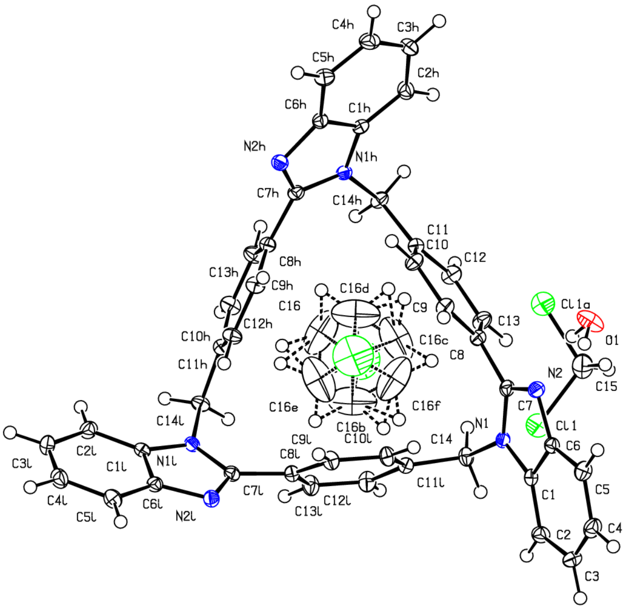

Figure S16. ORTEP drawing of *syn*-**2** at the 50% probability level.

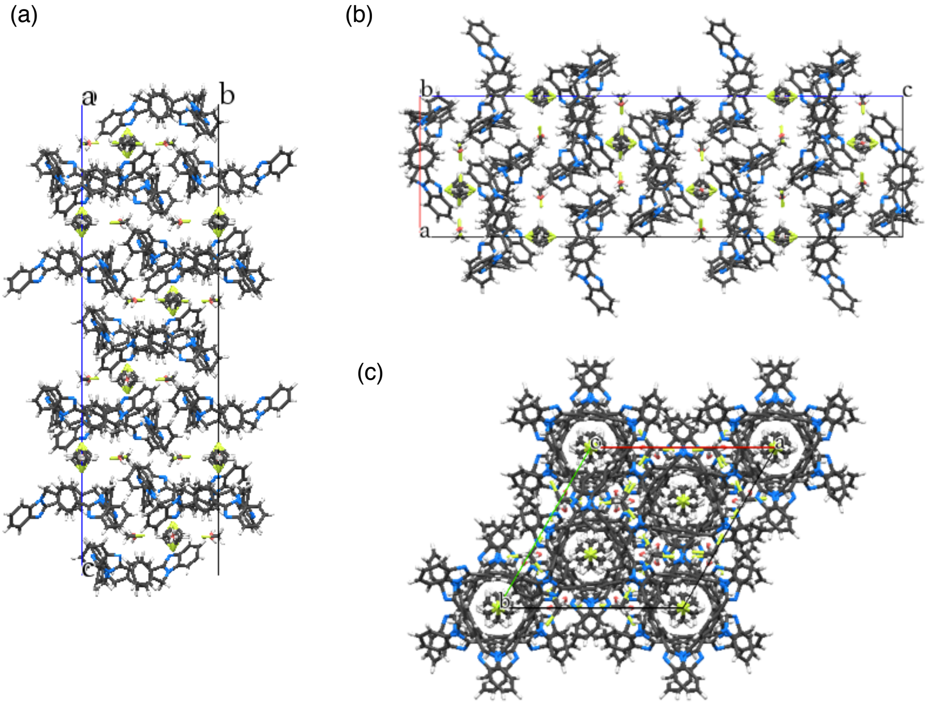

Figure S17. Unit cell structures of *syn*-**2** along (a) *a*-, (b) *b*-, and (c) *c*-axes.

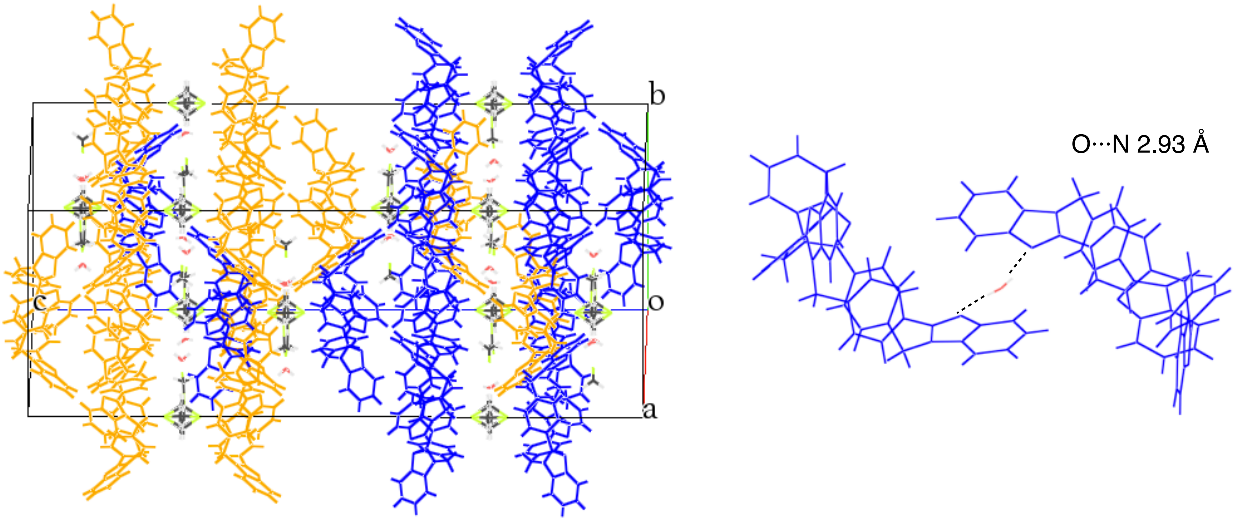

Figure S18. The positions of the (*P*)-isomer (orange) and the (*M*)-isomer (blue) of *syn*-**2** in the unit cell, and water-mediated hydrogen bonding of *syn*-**2**.

Pale yellow crystals of *anti*-**3** suitable for the single-crystal XRD analysis were obtained by recrystallization of a mixed solution of *anti*-**3** in $CHCl_3$/acetone = 1/1.

Crystal data for *anti*-**3**·$(CHCl_3)_2$: $C_{44}H_{32}Cl_6N_6$, F_w = 857.45, crystal dimensions 0.25 × 0.10 × 0.10 mm, monoclinic, space group *Cc*, a = 5.7565(5), b = 25.735(2), c = 26.340(2) Å, β = 91.020(2)°, V = 3901.4(6) Å^3, Z = 4, ρ_{calcd} = 1.460 g cm^{-3}, μ = 0.483 mm^{-1}, T = 93 K, λ(MoKα) = 0.71075 Å, $2\theta_{max}$ = 51.0°, 15995/6373 reflections collected/unique (R_{int} = 0.0546), R_1 = 0.0911 ($I > 2\sigma(I)$), wR_2 = 0.2893 (for all data), GOF = 1.060, largest diff. peak and hole 0.796/–0.686 eÅ^{-3}. CCDC deposit number 1585799.

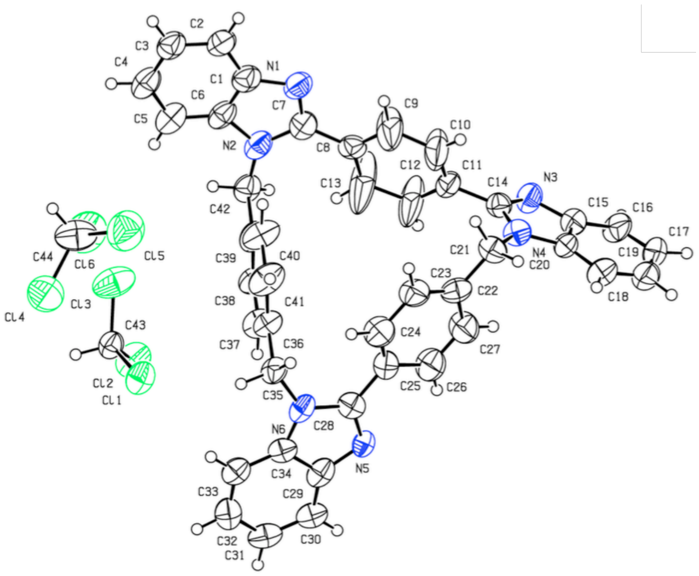

Figure S19. ORTEP drawing of *anti*-**3** at the 50% probability level.

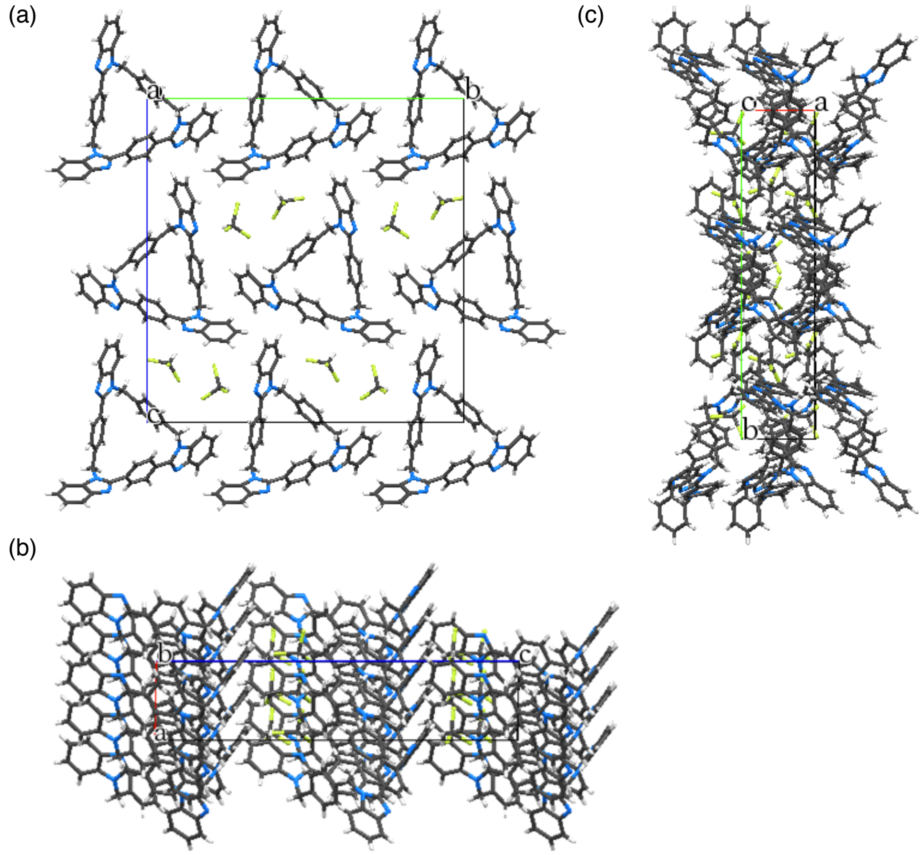

Figure S20. Unit cell structures of *anti*-**3** along (a) *a*-, (b) *b*-, and (c) *c*-axes.

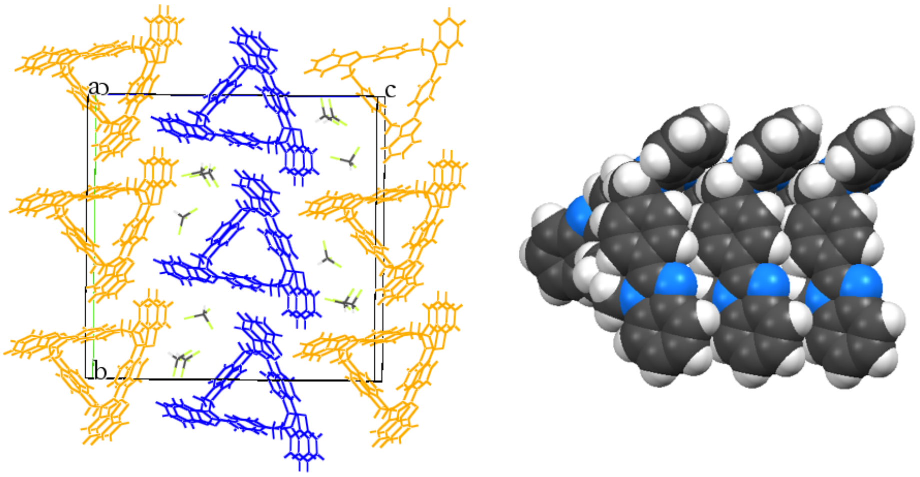

Figure S21. The positions of the (*P*)-isomer (orange) and the (*M*)-isomer (blue) of *anti*-**3** in the unit cell, and a stacking structure of *anti*-**3**.

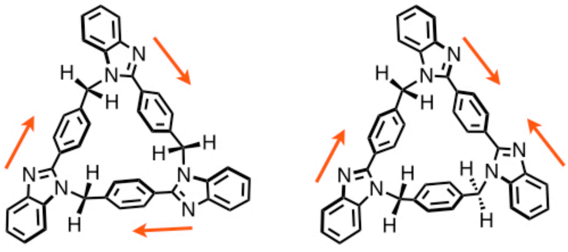

(*P*)-*syn*-**2** (*P*)-*anti*-**3**

viewed from the bottoms

Figure S22. Definition of the (*P*)- and (*M*)-helical isomers of *syn*-**2** and *anti*-**3**.

Variable-temperature ^{1}H NMR of *syn*-**2** and *anti*-**3**

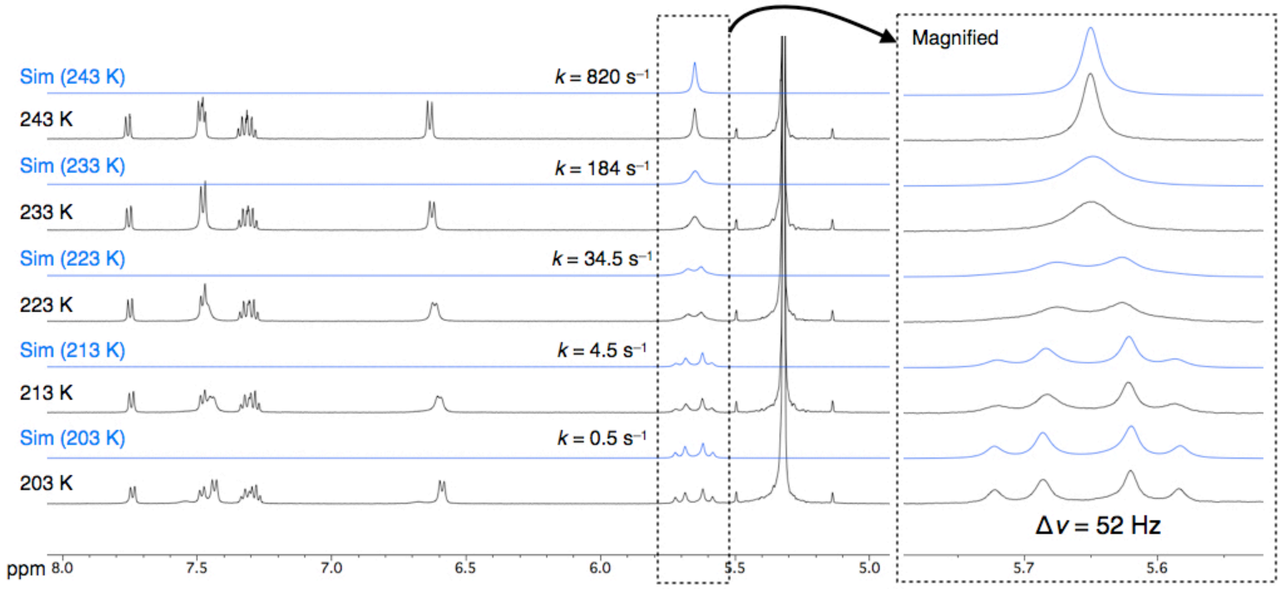

Figure S23. VT-^{1}H NMR spectra and line-shape analyses of *syn*-**2** (500 MHz, CD_2Cl_2, [*syn*-**2**] = 0.5 mM). Black and blue lines show observed and simulated spectra, respectively, at each temperature. In the dynamic line-shape simulations, not only the main AB spin system at 5.60 and 5.70 ppm but also another small spin system at around 5.64 ppm were considered because a minor conformational isomer was observed at 6.68 and 7.55 ppm at 203 K.

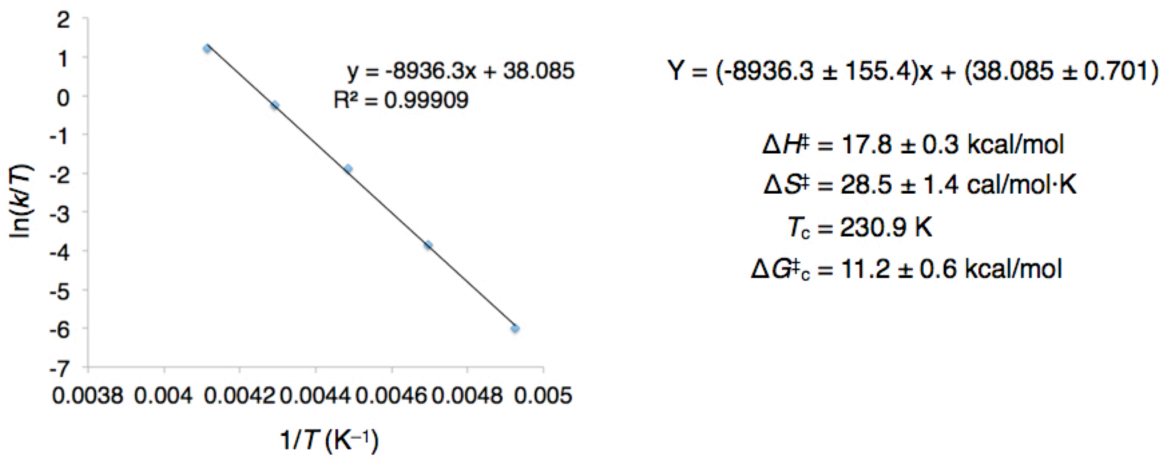

Figure S24. Eyring plot for *syn*-**2** in CD_2Cl_2 based on the result of Figure S24. $\Delta H^{\ddagger} = -R \times$ (slope), $\Delta S^{\ddagger} = R \times [(y \text{ intercept}) - \ln(k_b/h)]$, $\Delta G^{\ddagger}{}_{c} = \Delta H^{\ddagger} - T_c\Delta S^{\ddagger}$, R = 8.314 J/K·mol, $k_b = 1.38 \times 10^{-23}$ J/K, and $h = 6.63 \times 10^{-34}$ J·s.

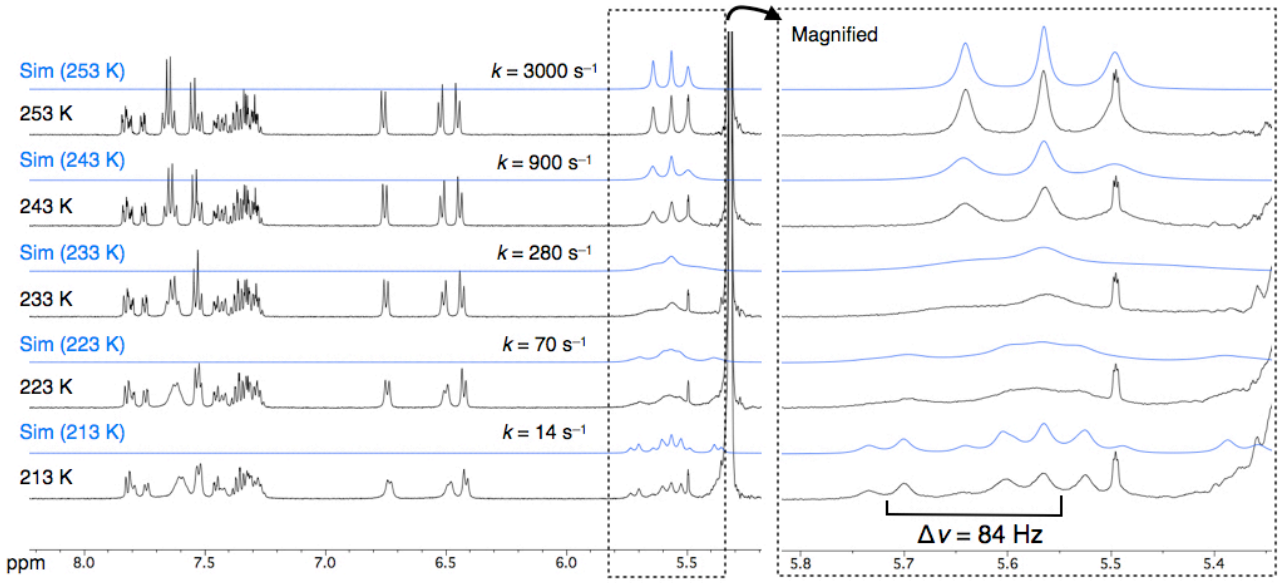

Figure S25. VT ^{1}H NMR spectra and line-shape analyses of *anti*-**3** (500 MHz, CD_2Cl_2, [*anti*-**3**] = 0.6 mM). Black and blue lines show observed and simulated spectra, respectively, at each temperature. In the dynamic line-shape simulations, three AB spin systems were considered.

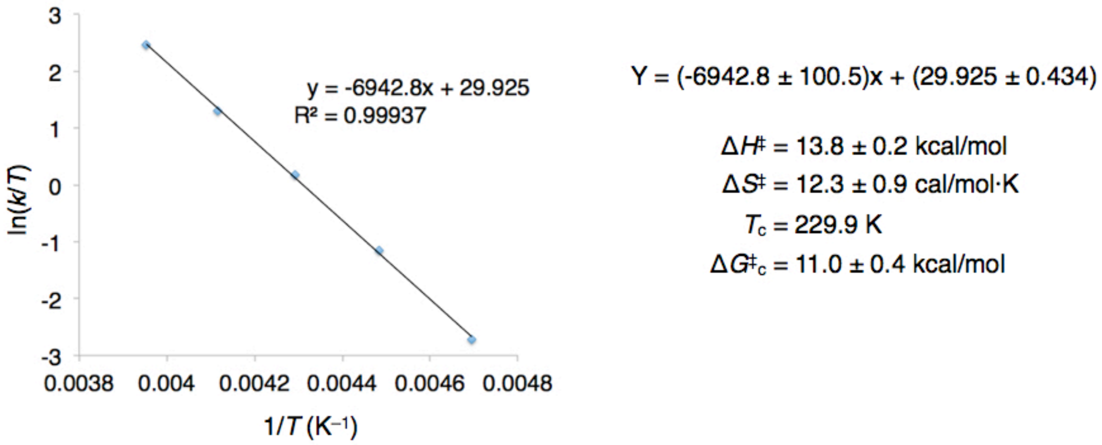

Figure S26. Eyring plot for *anti*-**3** in CD_2Cl_2 based on the result of Figure S25. $\Delta H^{\ddagger} = -R \times$ (slope), $\Delta S^{\ddagger} = R \times [(y \text{ intercept}) - \ln(k_b/h)]$, $\Delta G^{\ddagger}_c = \Delta H^{\ddagger} - T_c\Delta S^{\ddagger}$, R = 8.314 J/K·mol, $k_b = 1.38 \times 10^{-23}$ J/K, and $h = 6.63 \times 10^{-34}$ J·s.

Methylation of *anti*-3

As a demonstration of the chemical modification of benzimidazole[3]arenes, methylation of *anti*-**3** was examined as below. On the other hand, we have not yet examined any modification of *syn*-**2**.

Compound *anti*-**3** (0.15 mg, 0.24 μmol) was dissolved in $CHCl_3$ (0.1 mL), and mixed with an excess amount of CH_3I (*ca.* 2000 equiv.) at room temperature. After 1 week, the reaction mixture became yellow suspension, but the reaction did not complete. The solvent was changed into

acetonitrile to dissolve the suspension, and a further reaction with excess CH_3I was conducted at room temperature for 10 days to complete the tri-methylation of *anti*-**3**.

Note that the inversion of methylated *anti*-**3** was still faster than the NMR timescale at 300 K as shown in Figure S27. To slow the inversion rate down, we tried to modify *anti*-**3** with a longer alkyl halide (*n*-$C_{16}H_{33}Br$). However, the reaction was very slow and a part of alkylated products gradually decomposed under harsh conditions as far as we examined.

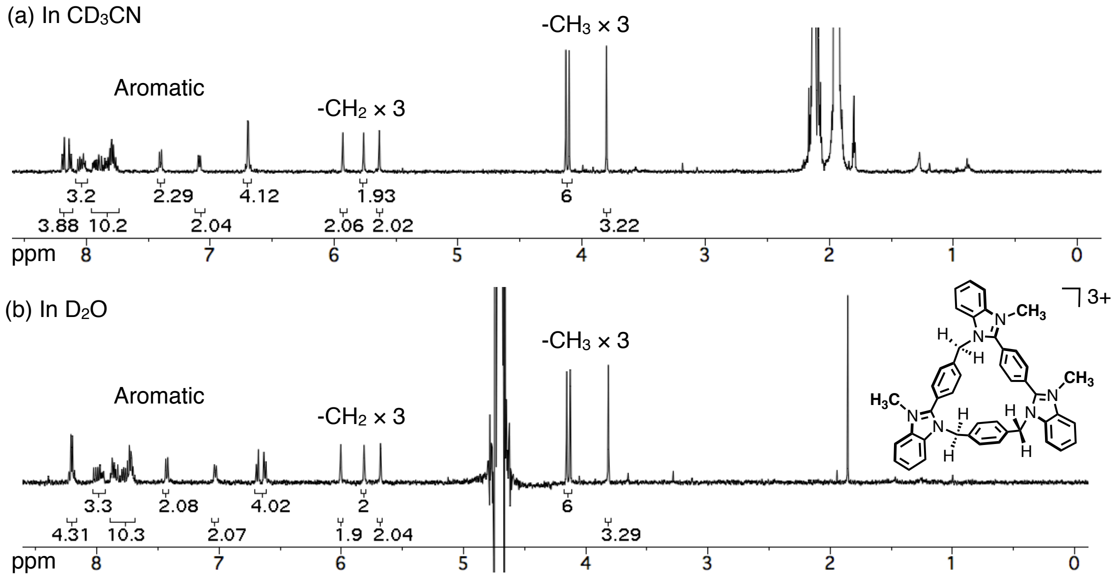

Figure S27. ^{1}H NMR spectra of tri-methylated *anti*-**3** (500 MHz, 300 K) in (a) CD_3CN and (b) D_2O.

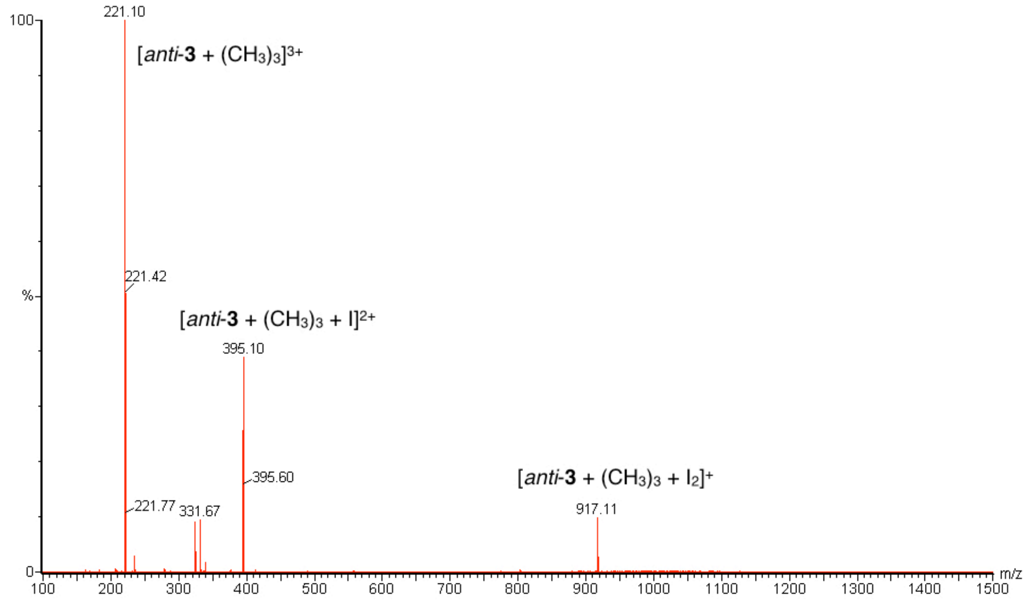

Figure S28. ESI-TOF mass spectrum of tri-methylated *anti*-**3** (positive, CH_3CN).

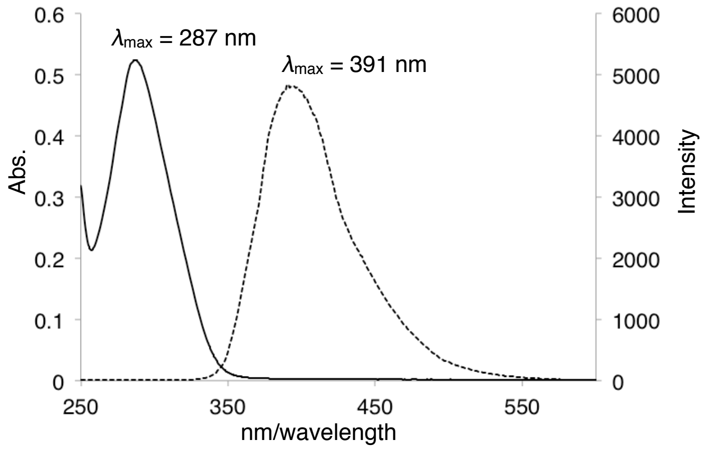

Figure S29. UV-vis absorption (solid line) and luminescence (dash line) spectra of tri-methylated *anti*-**3** in water (H_2O, 298 K, λ_{ex} = 289 nm).

Protonation of *anti*-3

To the solution of *anti*-**3** (0.21 mg, 0.34 µmol) in CD_2Cl_2 (0.6 mL) was added *p*-toluenesulfonic acid monohydrate (*p*-TsOH·H_2O) (8 equiv.) at room temperature. After the slow evaporation of the solution for several weeks, colorless plates appeared, and one of them was analyzed by single-crystal XRD analysis.

Crystal data for *anti*-**3**·(*p*-TsOH)$_3$·(H_2O)$_3$: $C_{63}H_{60}N_6O_{12}S_3$, F_w = 1189.35, crystal dimensions 0.079 × 0.043 × 0.028 mm, triclinic, space group *P*–1, a = 9.9758(3), b = 14.1235(3), c = 21.6617(4) Å, α = 98.632(2), β = 94.107(2), γ = 107.170(2)°, V = 2861.14(12) Å^3, Z = 2, ρ_{calcd} = 1.381 g cm^{-3}, μ = 1.768 mm^{-1}, T = 93 K, λ(CuKα) = 1.54184 Å, $2\theta_{max}$ = 148.9°, 32111/11270 reflections collected/unique (R_{int} = 0.0433), R_1 = 0.0488 ($I > 2\sigma(I)$), wR_2 = 0.1332 (for all data), GOF = 1.047, largest diff. peak and hole 0.450/–0.558 eÅ^{-3}. CCDC deposit number 1585801.

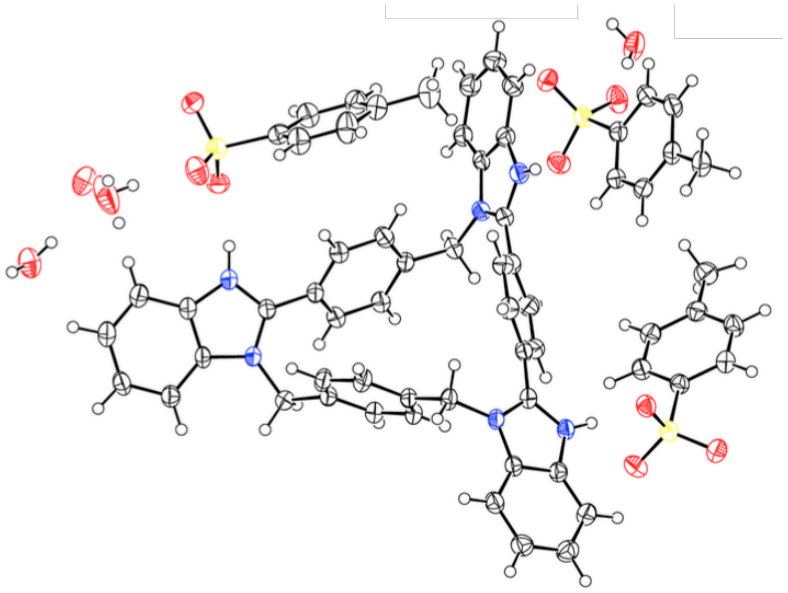

Figure S30. ORTEP drawing of *anti*-**3**·(*p*-TsOH)$_3$·(H_2O)$_3$ at the 50% probability level.

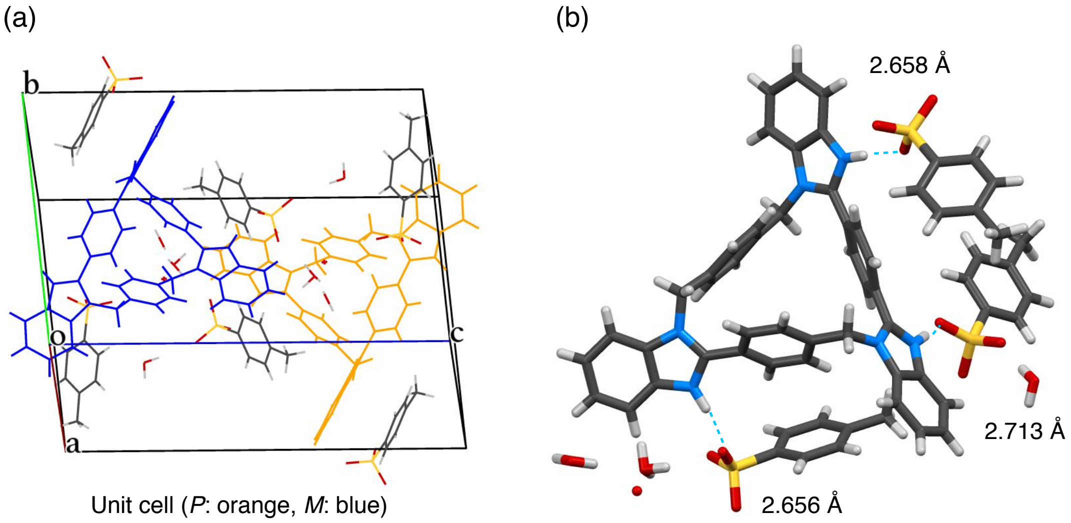

Figure S31. (a) Unit cell structure of *anti*-**3**·(*p*-TsOH)$_3$·(H_2O)$_3$ and (b) hydrogen bonding between a protonated nitrogen atom and a sulfonate oxygen atom (distances between N and O).

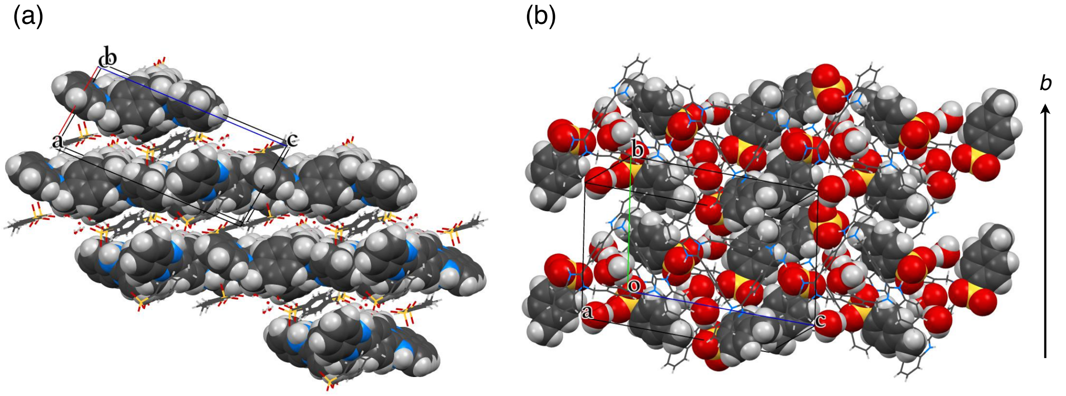

Figure S32. (a) Layer-by-layer structure composed of the macrocycle layers of *anti*-**3** and the hydrated layers of *p*-TsO$^-$ anions and (b) hydrogen bond network of water molecules and *p*-TsO$^-$ anions along *b*-axis.

Formation of triimine intermediates 2’ and 3’

^{1}H NMR analyses

Tris(*o*-phenylenediamine) macrocycle **1** (0.15 mg, 0.24 μmol, 1 equiv) was dissolved in CD_3CN (*ca.* 0.5 mL) by heating. To the solution was added $Cu(OAc)_2 \cdot H_2O$ (0.15 mg, 0.75 μmol, 3.1 equiv) dissolved in CD_3CN (*ca.* 0.2 mL). The reaction at room temperature was monitored by ^{1}H NMR analyses. After 8 days, the molar ratio of *syn*-**2** and *anti*-**3** was estimated to be *ca.* 1:10 based on the integral ratios.

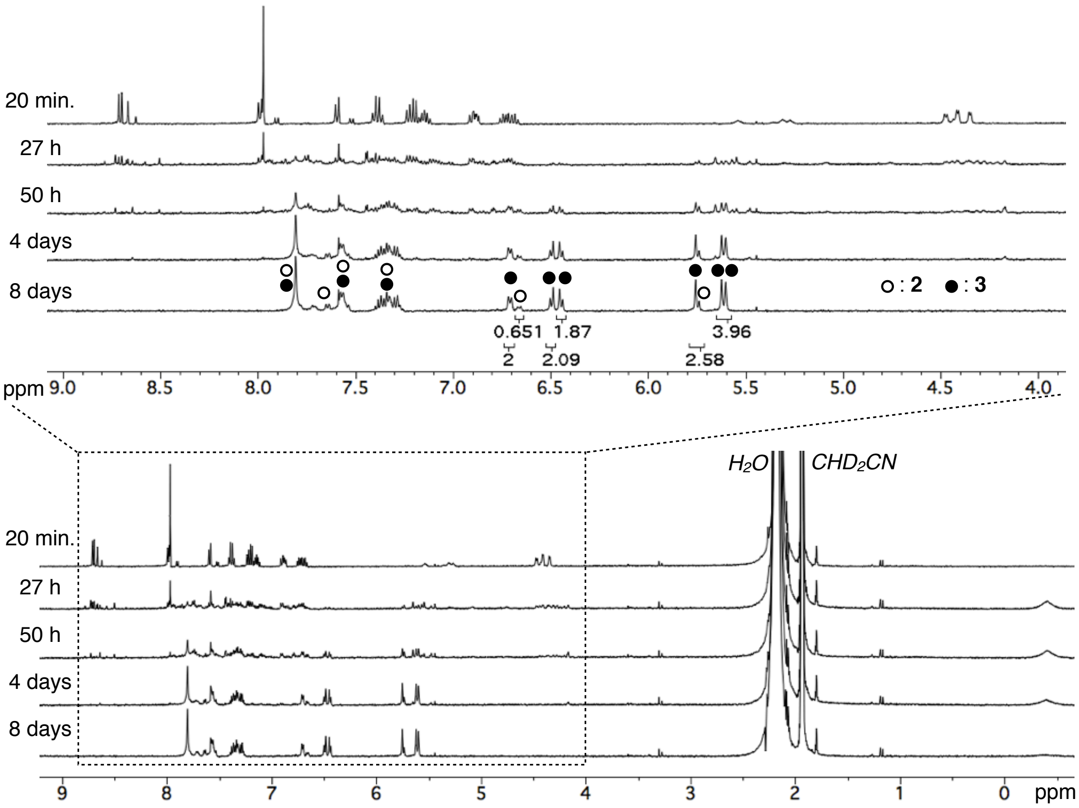

Figure S33. ^{1}H NMR spectra of the reaction (500 MHz, CD_3CN, 300 K) in which the reaction times are shown on the left of each spectrum.

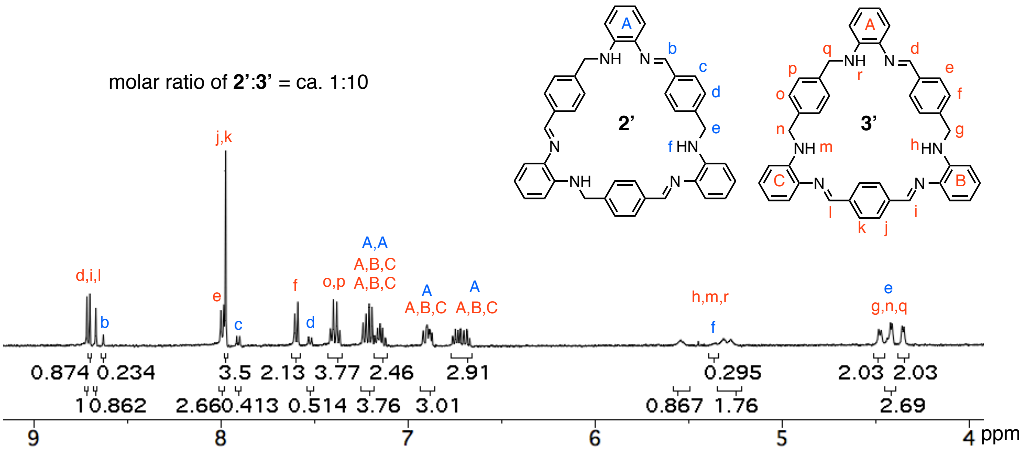

Figure S34. Partial ^{1}H NMR spectrum of the reaction solution after 20 min (500 MHz, CD_3CN, 300 K).

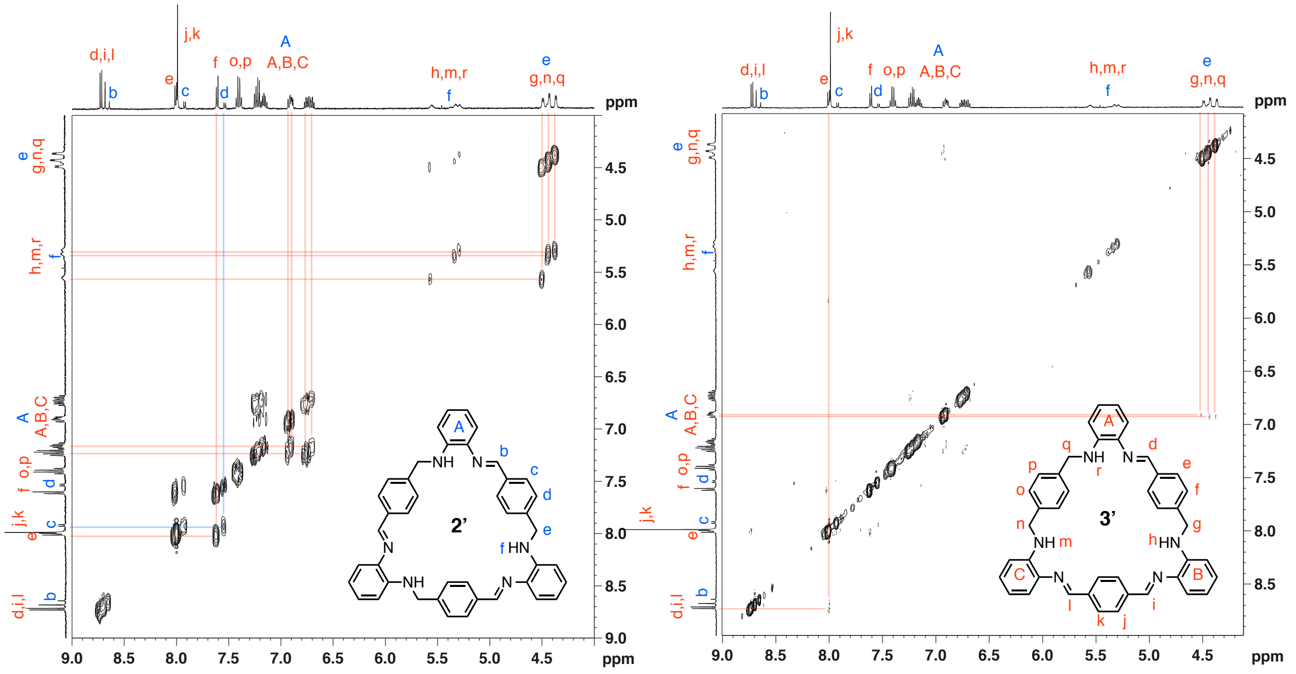

Figure S35. ^{1}H-^{1}H COSY (left) and ^{1}H-^{1}H NOESY (right, mixing time = 0.3 s) of the reaction solution after 20 min (500 MHz, CD_3CN, 300 K).

ESI-TOF mass analyses

Tris(*o*-phenylenediamine) macrocycle **1** (0.26 mg, 0.41 μmol, 1 equiv) was dissolved in CH_3CN (0.9 mL) by heating. A part of this solution was first analyzed by ESI-TOF mass spectrometry. To the solution was added $Cu(OAc)_2 \cdot H_2O$ (0.28 mg, 1.4 μmol, 3.4 equiv) dissolved in CH_3CN (0.4 mL). After 20 min at room temperature, the resulting yellow solution was passed through silica gel and a glass fiber filter to remove copper species and then analyzed by ESI-TOF mass spectrometry again.

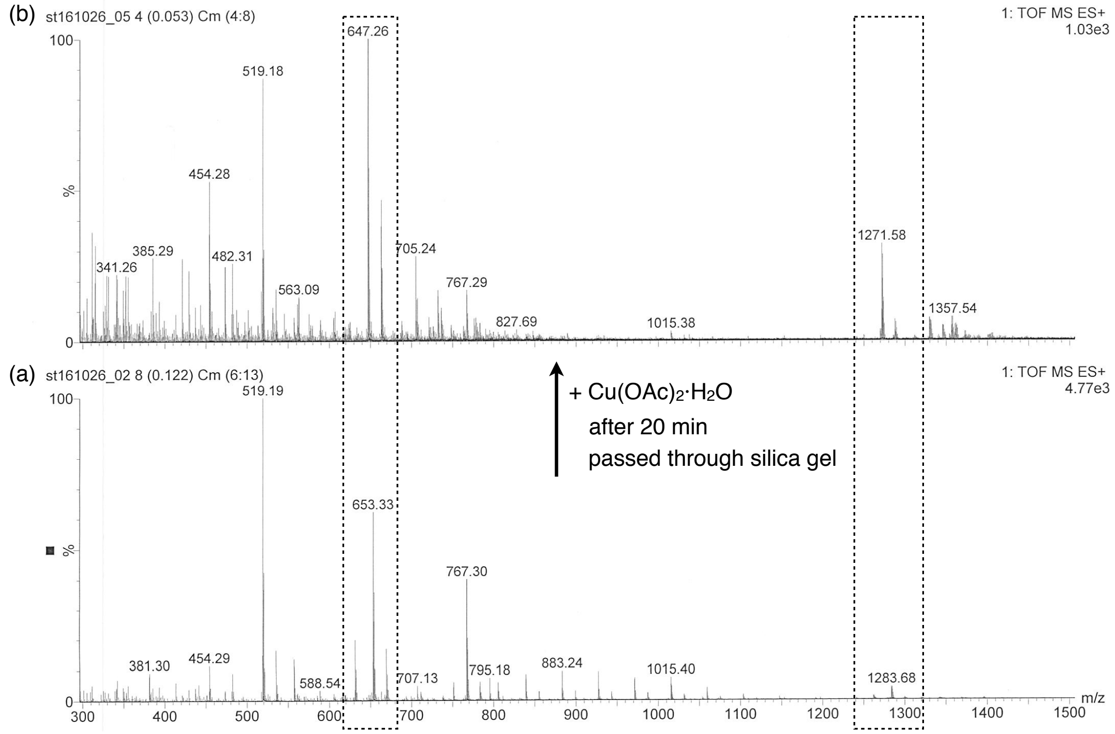

Figure S36. ESI-TOF mass spectra (positive mode, CH_3CN) of **1** (a) before mixing with $Cu(OAc)_2 \cdot H_2O$ and (b) after the reaction for 20 min (Cu species were removed). The two regions surrounded by dash lines are magnified on Figures S37 and S38.

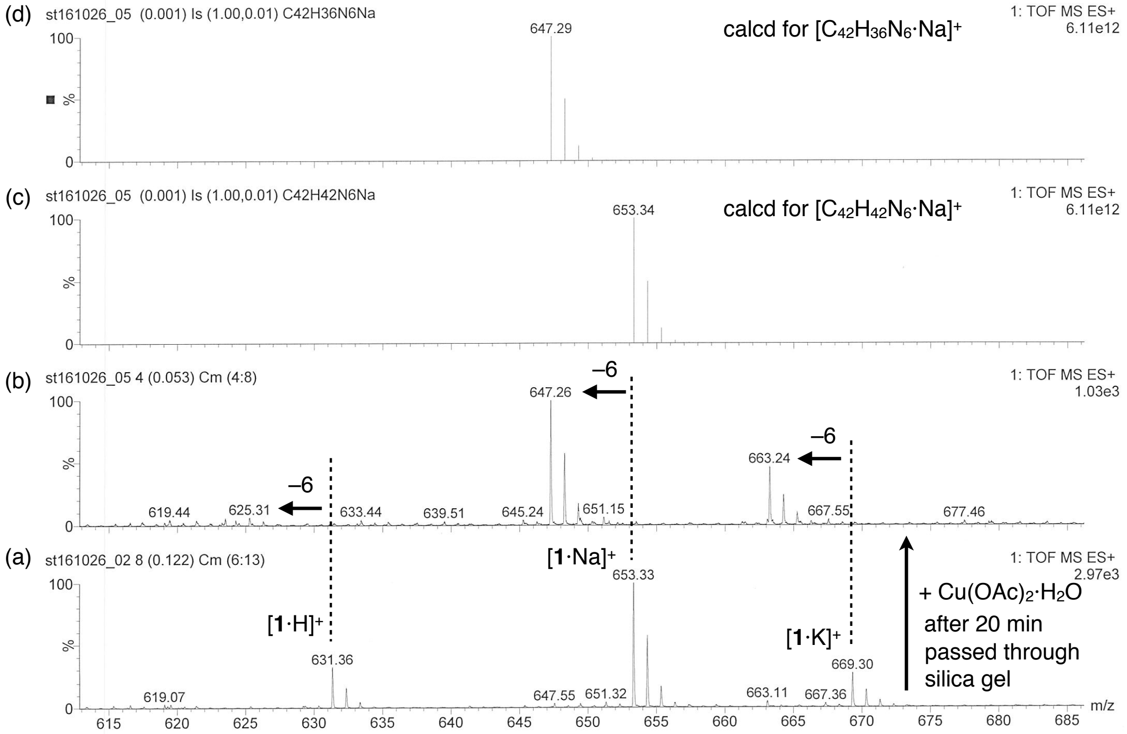

Figure S37. Magnified ESI-TOF mass spectra of Figure S36. (a) Before mixing with $Cu(OAc)_2 \cdot H_2O$, (b) after the reaction for 20 min (Cu species were removed), (c) and (d) theoretical spectra.

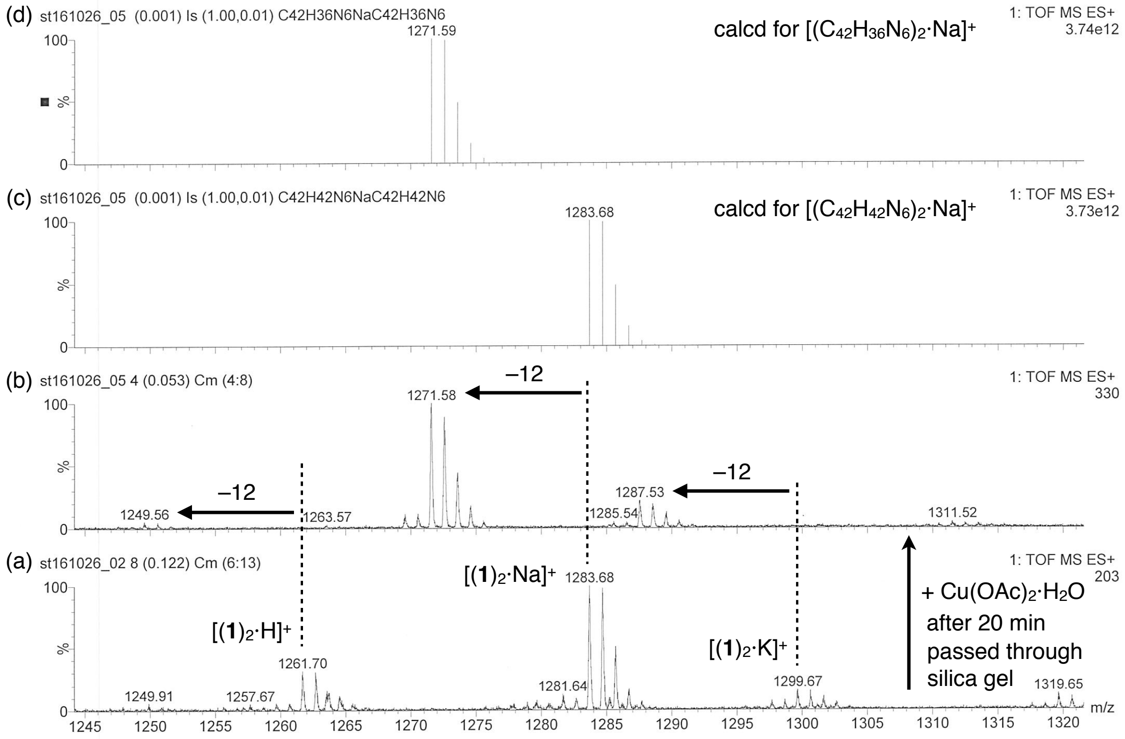

Figure S38. Magnified ESI-TOF mass spectra of Figure S36. (a) Before mixing with $Cu(OAc)_2{\cdot}H_2O$, (b) after the reaction for 20 min (Cu species were removed), (c) and (d) theoretical spectra.

Reaction at higher temperature without $Cu(OAc)_2 \cdot H_2O$

In a vial, tris(*o*-phenylenediamine) macrocycle **1** (0.22 mg, 0.35 μmol) was suspended in CD_3CN (0.6 mL) under air. The vial was capped and placed in a microwave cavity. Microwave was then irradiated at 180 °C with stirring at 600 rpm. The reaction was monitored by ^1H NMR analyses.

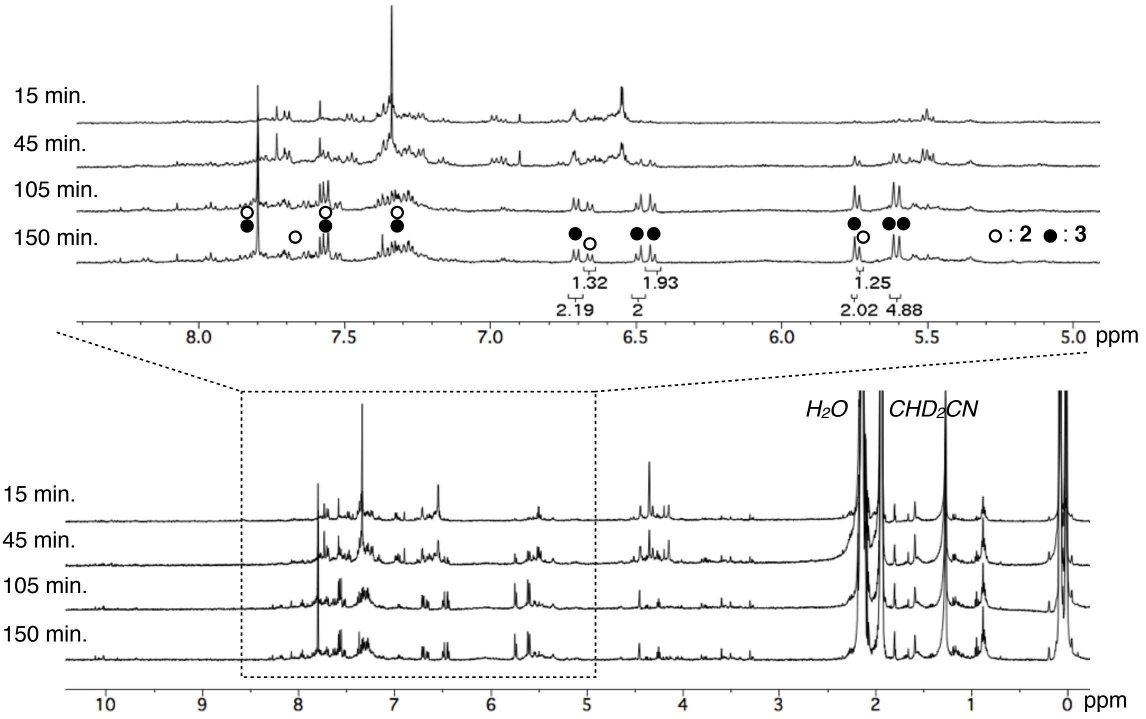

Figure S39. ^1H NMR spectra of the reaction solution (500 MHz, CD_3CN, 300 K) in which the reaction times are shown on the left of each spectrum.

Reaction with DDQ

Tris(*o*-phenylenediamine) macrocycle **1** (0.37 mg, 0.59 μmol, 1.0 equiv) was dissolved in CD_3CN (0.6 mL) by heating. To the solution was added a CD_3CN solution (0.1 mL) dissolving 2,3-dichloro-5,6-dicyanobenzoquinone (DDQ) (0.77 mg, 3.4 μmol, 5.8 equiv). The solution immediately turned red at room temperature. After 4 h, DDQ (0.73 mg, 3.2 μmol, 5.4 equiv) was added to the solution again and the solution was left to stand at room temperature for 3 weeks. The resultant solution was roughly purified by silica gel column chromatography to afford a crude product as a pale yellow solid, which was then analyzed by ^1H NMR spectroscopy.

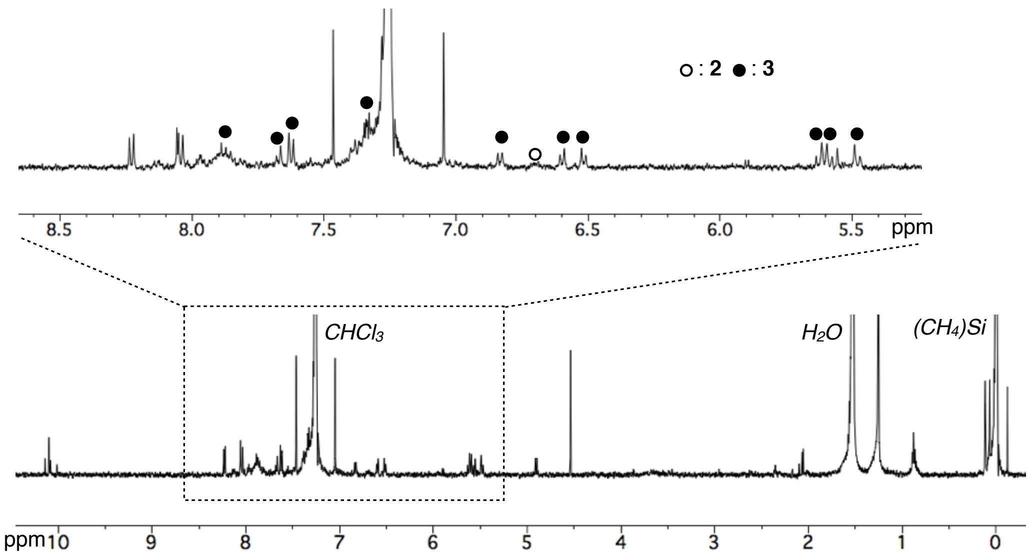

Figure S40. ^1H NMR spectra of the crude product (500 MHz, $CDCl_3$, 300 K).

References

[1] S. Tashiro, R. Kubota and M. Shionoya, *J. Am. Chem. Soc.*, 2012, **134**, 2461–2464.

[2] G. M. Sheldrick, *SHELXL-97, Program for refinement of crystal structure* (University of Göttingen, Göttingen, Germany, 1997); SHELXL-2013 (University of Göttingen, Göttingen, Germany, 2013).
